# RAGE inhibition blunts insulin-induced oncogenic signals in breast cancer

**DOI:** 10.1186/s13058-023-01686-5

**Published:** 2023-07-17

**Authors:** M. G. Muoio, M. Pellegrino, V. Rapicavoli, M. Talia, G. Scavo, V. Sergi, V. Vella, S. Pettinato, M. G. Galasso, R. Lappano, D. Scordamaglia, F. Cirillo, A. Pulvirenti, D. C. Rigiracciolo, M. Maggiolini, A. Belfiore, E. M. De Francesco

**Affiliations:** 1 Endocrinology, Department of Clinical and Experimental Medicine, Garibaldi-Nesima Hospital, University of Catania, 95122 Catania, Italy; 2 Department of Pharmacy, Health and Nutritional Sciences, University of Calabria, 87036 Rende, Italy; 3 Breast Unit Breast Surgery, Garibaldi-Nesima Hospital, 95122 Catania, Italy; 4 Pathological Anatomy Unit, Garibaldi-Nesima Hospital, 95122 Catania, Italy; 5 Bioinformatics Unit, Department of Clinical and Experimental Medicine, University of Catania, 95131 Catania, Italy; 6 Department of Experimental Oncology, IEO, European Institute of Oncology IRCCS, Via Adamello 16, 20139 Milan, Italy

**Keywords:** Insulin, Insulin receptor, Breast cancer, RAGE, CAFs

## Abstract

The receptor for advanced glycation end products (RAGE) is implicated in diabetes and obesity complications, as well as in breast cancer (BC). Herein, we evaluated whether RAGE contributes to the oncogenic actions of Insulin, which plays a key role in BC progression particularly in obese and diabetic patients. Analysis of the publicly available METABRIC study, which collects gene expression and clinical data from a large cohort (*n* = 1904) of BC patients, revealed that *RAGE* and the Insulin Receptor (*IR*) are co-expressed and associated with negative prognostic parameters. In MCF-7, ZR75 and 4T1 BC cells, as well as in patient-derived Cancer-Associated Fibroblasts, the pharmacological inhibition of RAGE as well as its genetic depletion interfered with Insulin-induced activation of the oncogenic pathway IR/IRS1/AKT/CD1. Mechanistically, IR and RAGE directly interacted upon Insulin stimulation, as shown by in situ proximity ligation assays and coimmunoprecipitation studies. Of note, RAGE inhibition halted the activation of both IR and insulin like growth factor 1 receptor (IGF-1R), as demonstrated in MCF-7 cells KO for the *IR* and the *IGF-1R* gene via CRISPR-cas9 technology. An unbiased label-free proteomic analysis uncovered proteins and predicted pathways affected by RAGE inhibition in Insulin-stimulated BC cells. Biologically, RAGE inhibition reduced cell proliferation, migration, and patient-derived mammosphere formation triggered by Insulin. In vivo, the pharmacological inhibition of RAGE halted Insulin-induced tumor growth, without affecting blood glucose homeostasis. Together, our findings suggest that targeting RAGE may represent an appealing opportunity to blunt Insulin-induced oncogenic signaling in BC.

**Supplementary Information:**

The online version contains supplementary material available at 10.1186/s13058-023-01686-5.

## Introduction

Breast cancer (BC) risk and mortality are typically higher in conditions of obesity and diabetes, metabolic disorders associated with inflammation and de-regulated action of Insulin (Ins) [1–3]. In fact, along with the well-known role in metabolic regulation, Ins elicits stimulatory responses that contribute to the acquisition of malignant features in diverse tumors, including BC [4]. The tumor-promoting effects of Ins are mainly mediated by the Insulin Receptor (IR), a receptor tyrosine kinase (RTK) expressed in almost 80% of BCs and associated with worse prognostic outcomes [5, 6]. Ins binds to IR and triggers its phosphorylation, leading to the activation of Insulin Receptor Substrate (IRS) proteins, and the subsequent engagement of PI3K/AKT and MAPK signaling pathways, toward gene expression changes and biological responses [7]. Ins/IR-initiated signals induce cancer cell survival, proliferation, migration, invasion, angiogenesis, metabolic reprogramming, acquisition of stemness features, and therapeutic resistance [8], thus fostering disease progression. It should be mentioned that beyond Ins, IR may act as a receptor for both Insulin-like Growth Factor (IGF)-1 (IGF-1) and IGF-2, which themselves prompt tumor-promoting responses [9]. Not surprisingly, Ins, IGF-1, IGF-2 and their receptors (IR, IGF-1R and IGF-2R) and binding proteins (IGFBPs) constitute the Ins/IGF system (IIGFs), a critical signaling network well-dissected for its role in malignant progression also in BC [10, 11]. Notwithstanding the critical role of IR in cancer, poor benefit has been gained from targeting Ins-induced signaling and its network of transduction companion [12], thus calling for further research efforts.

The Receptor for Advanced Glycation End Products (RAGE) is a single-spanning multi-ligand membrane protein belonging to the superfamily of immunoglobulin (Ig) receptors and mainly implicated in the regulation of innate immunity and inflammation [13]. Notably, RAGE-mediated actions contribute to certain metabolic and inflammatory traits that characterize obesity, diabetes and cancer [14]. Despite the relatively recent identification of this receptor, RAGE-interfering agents, newly synthetized and/or repurposed, are attracting great basic and clinical research interest for their therapeutic potential in inflammatory and hyperproliferative disorders [15].

We have recently assessed that RAGE inhibition obliterates the angiogenic responses triggered by IGF-1/IGF-1R in BC [16]. These findings have outlined a previously unidentified cross-talk between IGF-1R and RAGE in breast malignancies.

In the present study, we demonstrate that RAGE cooperates also with IR in Ins-rich BC environments, facilitating Ins-induced oncogenic responses. Our findings further dissect the complexity of Ins/IR signaling and suggest that RAGE may serve as a novel therapeutic target for a better control of BC, particularly in patients affected by obesity and diabetes.

## Materials and methods

### Reagents

Ins, RAP and *N*-Acetyl-Cysteine were from Sigma-Aldrich (Italy). IGF-1 and IGF-2 were purchased from PeproTech (UK). FPS-ZM1, Linsitinib (OSI-906), NT157 and MK-2206 were purchased from Selleck Chemicals (distributed by Aurogene, Italy). IGF-1 and IGF-2 were solubilized in 10 mM Acetic Acid; Ins was dissolved in 0.01 M HCl; RAP and NAC were solubilized in DNase/RNase free water, while FPS-ZM1, OSI-906, NT157 and MK-2206 were dissolved in DMSO.

### Publicly available molecular datasets

Bioinformatics analyses were performed on R Studio (version 3.6.1) using the publicly available dataset Molecular Taxonomy of Breast Cancer International Consortium (METABRIC) [17]. The clinical information and the microarray mRNA expression data (Log2 transformed intensity values) of the METABRIC cohort were retrieved from cBioPortal for Cancer Genomics (http://www.cbioportal.org/) on December 18th 2022. Pearson correlation coefficient (r-value) was calculated using the R *cor.test* function and setting the method as “Pearson”. Box plots and scatter plots were assessed with the R *tidyverse* package. The statistical analyses were performed by using the t-test.

### Survival analysis

The survival analyses on BC patients were assessed using RAGE and IR gene expression data of the METABRIC dataset. Samples (*n* = 1904) were filtered for missing values and the vital status. As patients classified as “died of other causes” were excluded, we referred to “breast cancer specific survival” (BCS) (*n* = 1423). The survival analysis on RAGE expression has been performed using the survivAL package [18] through which we examined Cox proportional hazards for all possible points‐of‐separation (low‐high cut‐points), thus dividing the samples with high (*n* = 1186) and low (*n* = 213) RAGE expression levels according to the most significant cut-point. Furthermore, RAGE and IR survival analysis was carried out analyzing the breast cancer specific survival and dividing the patients into low expression of RAGE and IR (N = 1215, lower quartiles Q1 to Q3) or high expression of RAGE and IR expression (N = 175, upper quartile Q4). A log-rank test was used to determine differences between the survival curves. The Kaplan–Meier survival curves were generated using the survival and the survminer R packages. *P* < 0.05 was considered statistically significant.

### Cell cultures

MCF-7 and ZR75 BC cells, obtained from ATCC, were maintained in MEM (Sigma-Aldrich, Italy) supplemented with 10% fetal bovine serum (FBS), 1% penicillin/streptomycin (P/S), 1% MEM Non-Essential Amino Acids and 1% Glutamine (Thermo Fisher Scientific, Life Technologies, Italy). 4T1 cells, obtained from ATCC, were maintained in RPMI-1640 (Sigma-Aldrich, Italy) supplemented with 10% FBS and 1% P/S. 293Ta packaging cells, obtained from Genecopoeia (distributed by Tebu-Bio, Italy), were maintained in high glucose DMEM supplemented with 10% FBS, 1% P/S and 1% Glutamax.

MCF-7 Clustered Regularly Interspaced Short Palindromic Repeats (CRISPR)-cas9, knock-out (KO)-IGF-1R and knock-out (KO)-IR were purchased from Applied Biological Materials (Richmond, BC, Canada). MCF7-RAGE cells, which overexpress the human RAGE protein (MCF7-RAGE) and the negative control (MCF7-Ex-Neg), were generated by lentiviral transduction (see below). Cancer-Associated Fibroblasts (CAFs) were obtained from six patients affected by invasive mammary ductal carcinoma undergoing mastectomy, as previously described [19] (see paragraph “Human breast tumor samples collection”). A detailed protocol for CAFs isolation, characterization and maintenance is provided in Supplementary Materials and Methods. All cell lines were grown in a 37 °C incubator with 5% CO_2_. Cells were switched to 1% charcoal-treated (CT) the day before the experiment.

### Lentiviral gene transduction

A stable RAGE-overexpressing MCF-7 cell line (MCF7-RAGE) was generated by lentiviral gene transduction, using packaging cells, reagents and lentiviral plasmids from Genecopoeia (distributed by Tebu-Bio, Italy), following a previously described protocol [16], as detailed in Supplementary Materials and Methods.

### Gene silencing

For knocking down RAGE expression, cells were seeded in six-well multi-dishes and transiently transfected with Lipofectamine RNAiMAX (Thermo Fisher Scientific, Life Technologies, Italy) using a mix of two siRNA targeting sequences (10 nM) (or scramble non-targeting control) (Origene, distributed by Tema Ricerca, Italy). Treatments were applied 24 h post-transfection.

### Gene expression studies

Total RNA from BC cells or tumor homogenates was extracted using TRIzol commercial kit (Thermo Fisher Scientific, Life Technologies, Italy), as recommended by manufacturer. Total cDNA was synthesized by reverse transcription, as previously described [19]. The expression of selected genes was quantified by real-time PCR using ABI 7500 Real-Time PCR System (Applied Biosystems, Thermo Fisher Scientific, Italy) with probe, primer sets and SYBR Green chemistry. Assays were performed in duplicate in at least two independent experiments. Results were normalized for 36B4 expression and then, calculated as fold induction of RNA expression. A detailed description of RNA extraction, reverse transcription qRT-PCR and primer sequences is available in Supplementary Materials and Methods.

### Human breast tumor samples collection and processing

BC surgical specimens were collected from patients undergoing surgery for primary BC removal at ARNAS Garibaldi-Nesima Hospital, Catania, Italy. Patient underwent fully informed consent, in accordance with local research ethics committee guidelines. The project was approved by local ethic committee. Tissues obtained were processed as previously described [19]. Briefly, samples were cut and placed in digestion solution overnight at 37 °C. Following digestion, samples were processed for BC cell isolation or CAFs isolation. For BC cells isolation, cells were strained first through a 70 μM filter (352,340, Corning, distributed by Sigma-Aldrich (Italy) and then, through a 40 μM filter (352,340, Corning, distributed by Sigma-Aldrich (Italy), each of which was rinsed with 3 × 1 ml MEM medium and centrifuged at 1000 g for 5 min at 4 °C to pellet cells. Supernatant was removed and the cell pellet resuspended in 100 μl of ice-cold PBS. Live cells were counted using Trypan Blue (Thermo Fisher Scientific, Life Technology, Italy), and cells were then cultured as mammospheres for 10 days (see below). CAFs isolation is described in Supplementary Materials and Methods.

### Western blot analysis

BC cells, CAFs and tumor homogenates were processed for total protein extraction as previously described [19]. Briefly, lysates were electrophoresed through polyacrylamide gels, electroblotted onto nitrocellulose membranes and probed with primary antibodies. Proteins were detected by horseradish peroxidase-linked secondary antibodies (Cell Signaling Technology, distributed by Euroclone, Italy) and revealed using the West Pico Chemiluminescent Substrate (Thermo Fisher Scientific, Life Technology, Italy). Chemiluminescent signal was revealed using Amersham high-performance chemiluminescence films (Hyperfilms Amersham, VWR, Italy), or the LI-COR Odyssey 2800 (Li-COR Inc., USA) and the software ImageStudioLite (version 5.2). A detailed description of protein extraction, Western blotting analysis and antibodies used is available in Supplementary Materials and Methods.

### Co-immunoprecipitation (Co-IP) assay

Co-IP assays were performed as previously described [20]. Briefly, after stimulation with treatments, cells were lysed using RIPA buffer (Sigma-Aldrich, Italy) containing protease (Merck Millipore, Italy) and phosphatase inhibitors (Thermo Fisher, Life Technologies, Italy). Protein lysates were clarified by centrifugation (14.000 rpm) for 15 min at 4 °C, and immunoprecipitation was carried out for 18 h. The immune complexes bound to protein G Sepharose (Sigma-Aldrich, Italy) were washed twice in lysis buffer and subjected to Western blot analysis. More details regarding Co-IP assays are available in Supplementary Materials and Methods.

### In situ proximity ligation assay (PLA)

PLA was performed using the Duolink kit (Sigma-Aldrich, Italy) to detect IR and RAGE direct interaction, as recommended by the manufacturer. A rabbit primary antibody against RAGE (D1A12, Cell Signaling Technology, distributed by Euroclone, Italy) and a mouse primary antibody against IR (L55B10, Cell Signaling Technology, distributed by Euroclone, Italy) were used, together with Anti-Rabbit PLUS and Anti-Mouse MINUS probes (PLA probes, Sigma-Aldrich, Italy). PLA signals were detected using a fluorescence microscope TI-E (Nikon, Netherland). More details are available in Supplementary Materials and Methods.

### Immunofluorescence assay

Fifty percent confluent CAFs were plated onto LabTek chamber slides (VWR, Italy) in regular growth medium for 24 h. Then, cells were fixed, permeabilized and incubated overnight with a primary antibody against FAPα (H-56, Santa Cruz Biotechnology, DBA, Italy). Thereafter, slides were washed and incubated with donkey anti-rabbit IgG-FITC or goat anti-rabbit IgG- Texas Red (Alexa Fluor, Thermo Fisher Scientific, Life Technologies, Italy) for 1 h at room temperature before mounting using Vectashield Antifade Mounting Medium with DAPI (DBA, Italy). A fluorescence microscope TI-E (Nikon, Netherland) was used to evaluate signals.

### Unbiased label-free semi-quantitative proteomics and pathway analysis

Proteomic analysis was carried out as previously described [21]. Protein lysates were then subjected to trypsin digestion, and peptides were prepared for LC–MS/MS analyses, which were performed on an LTQ Orbitrap XL mass spectrometer (Thermo Fisher Scientific, Life Technology, USA) coupled to an Ultimate 3000 RSLCnano system (Thermo Fisher Scientific, Life Technology, USA). Xcalibur raw data files acquired on the LTQ-Orbitrap XL were directly imported into Progenesis LCMS software (Waters Corp., UK) for peak detection and alignment. We considered as differentially expressed all proteins with an absolute Log2FC > 0.6 and a p-value ≤ 0.05, as calculated by ANOVA. Finally, DEPs were selected for MITHrIL pathway analysis [22]. More details are available in Supplementary Materials and Methods.

### Seahorse XFe-96 metabolic flux analysis

Extracellular acidification rates (ECAR) and oxygen consumption rates (OCR) were determined using the Seahorse Extracellular Flux (XFe-96) analyzer (Agilent Instruments, USA) as previously described [21]. Briefly, 15,000 MCF-7 cells were seeded into XF-96 cell culture microplates in regular growth medium. After 24 h, medium was switched to 1% CT in the presence of treatments. At the end of stimulation, cells were washed with pre-warmed XF assay medium (pH 7.4) (which was supplemented with 10 mM glucose, 1 mM Pyruvate, 2 mM L-glutamine for OCR measurements) and maintained in XF assay media at 37 °C, in a non-CO_2_ incubator for 1 h. During the incubation time, 5 μL of 80 mM glucose, 9 μM oligomycin, and 1 M 2-deoxyglucose (for ECAR measurement) or 10 μM oligomycin, 9 μM FCCP, 10 μM Rotenone, 10 μM antimycin A (for OCR measurement) (all purchased from Sigma-Aldrich, Italy) were loaded in XF assay media into the injection ports in the XFe-96 sensor cartridge. Data were analyzed using XFe-96 software. ECAR and OCR values were normalized by protein content, which was determined using Sulforhodamine (SRB) assay (see below).

### Cell proliferation

MCF-7 and ZR75 cells were seeded in six-well multi-dishes in complete medium. After 24 h, cells were washed and switched to phenol-red free MEM containing 1% CT, stimulated with treatments or transfected for 24 h (with siRNA RAGE or a scramble non-targeting control sequence) and then, stimulated with treatments. Cells were counted 72 h after treatments, using the Bright Line™ Hemacytometer (Sigma-Aldrich, Italy).

### Sulforhodamine B (SRB) Assay

Protein content in viable cells was assessed using the SRB assay. Briefly, after treatments cells were fixed with 10% trichloroacetic acid (TCA) (Sigma-Aldrich, Italy) for 1 h at 4 °C and then, dried overnight at room temperature. Then, cells were incubated with SRB (Sigma-Aldrich, Italy) for 15 min, washed twice with 1% acetic acid and air-dried for at least 1 h. Finally, the protein-bound dye was dissolved in a 10 mM Tris pH 8.8 solution and read using the plate reader Wallac Victor 1420 (PerkinElmer, USA) at 540 nm. As SRB stoichiometrically binds to proteins, the amount of bound dye was thereafter used as a proxy for cell mass and employed to extrapolate the rate of cell proliferation [23].

### Soft-agar colony formation assay

The bottom of six-well multi-dishes was plated with a mixture of 0.66% agar (Thermo Fisher Scientific, Life Technologies, Italy) and medium containing 5% CT (hard-agar). Then 2500 cells were suspended in 5% CT containing 0.33% agar (soft-agar) and plated on the top of the hard-agar layer. Cells were cultured in these conditions for three weeks in the presence or absence of treatments. Colonies were stained with 7 mg/mL of methyl thiazolyl tetrazolium (MTT) (Sigma-Aldrich, Italy), photographed, and analyzed using NIH ImageJ.

### Wound healing assay

70–80% confluent CAFs seeded in regular growth medium in 12-well plates were switched to medium without serum for 24 h, scratched using a p200 pipette tip and allowed to migrate into the wound in response to Ins, alone and in combination with FPS-ZM1. Pictures were taken at 0 h, 16 h after scratching, as indicated, using an inverted phase contrast microscope (5 × magnification). The rate of cell migration was measured by quantifying the % of wound closure area, determined using the software NIH ImageJ, according to the following formula:

$$\% {\text{ of wound closure}}\, = \,\left[ {\left( {{\text{At}}\, = \,0 {\text{h}}} \right){-}\left( {{\text{At}}\, = \,\Delta {\text{ h}}} \right)/\left( {{\text{At}}\, = \,0 {\text{h}}} \right)} \right]\, \times \,{1}00\% {\text{T}}$$

### Mammosphere assay

A single cell suspension of MCF-7 cells or BC patients-derived cells (obtained as described in the paragraph “Human breast tumor samples collection and processing”) was prepared using enzymatic (1 × Trypsin 0,25% EDTA (Thermo Fisher Scientific, Life Technologies, Italy) and manual disaggregation (25 gauge needle). Cells were then plated in mammosphere medium, composed of DMEM- F12, supplemented with insulin-free B27, and 1% P/S (all from Thermo Fisher Scientific, Life Technologies, Italy), together with EGF (20-ng/ml) (Sigma-Aldrich, Italy). Low-attachment, non-adherent culture plastics were generated by coating with (2-hydroxyethylmethacrylate) (poly-HEMA, Sigma-Aldrich, Italy). Treatments were added immediately after seeding for 5 days (in assays using MCF-7 cells) or 10 days (in assays using BC patients-derived cells). Next, spheres with diameter > 50 μm were counted, and the percentage of cells plated which formed spheres was calculated and is referred to as percentage of number of spheres compared to vehicle**-**treated cells. Mammosphere assays were performed in triplicate and repeated three times independently.

### Animal studies

Female 4-week-old athymic nude mice (nu/nu Swiss, Envigo, Italy) were maintained in a sterile environment. On day 0, 12,000 4T1 cells were implanted in the mammary fat pad in 0.150 ml Matrigel (R&D systems, distributed by BioTechne, Italy). After 1 week, tumors were detected, and mice were randomly allocated to four groups (*n* = 6) according to treatments administered for 28 days by subcutaneous injection of vehicle (NaCl) or Ins Glargine (5 days/week, 0.6 U/die), while FPS-ZM1 (1 mg/kg) was injected intraperitoneally 2 days/week. Mice were weighed every week, and blood glucose levels were acquired by a tail vein prick using a lancet and determined 15 min after treatments using a glucometer and glucose test strips (GlucoMen Areo Sensor, Menarini, Italy), according to the manufacturer’s instructions. 4T1 allograft tumor growth was measured twice a week by caliper, along two orthogonal axes: length (L) and width (W). Tumor volumes (in cubic centimeters) were estimated by the following formula: TV = L × [W2]/2. At 28 days of treatment, animals were sacrificed following the standard protocols, and tumors were dissected from the neighboring connective tissue. Tumor tissues were partly fixed in 4% formalin for 24 h prior to paraffin-embedding for subsequent histologic analyses and partly homogenated for RNA and proteins extraction, as described above. Animal studies were performed in accordance with the principles of the Declaration of Helsinki and the Italian law D.L. 26/2014. They were carried out also in accordance with the Guide for the Care and Use of Laboratory Animals of the US National Institutes of Health (2011), and the Directive 2010/63/EU of the European Parliament. Animal care, euthanasia, and experiments were performed according to the principle of the 3Rs (replacement, reduction, and refinement) and the institutional guidelines of the University of Calabria, Italy. The project was approved by the local ethical committee. Histologic analyses and immunohistochemistry were performed as described in Supplementary Materials and Methods.

### Statistical analysis

Differences between experimental groups were analyzed using ANOVA and independent *t* tests. *P* < 0.05 was considered statistically significant. Results are expressed as means ± SEM.

## Results

### IR and RAGE are co-expressed in BC patients

**Fig. 1:**
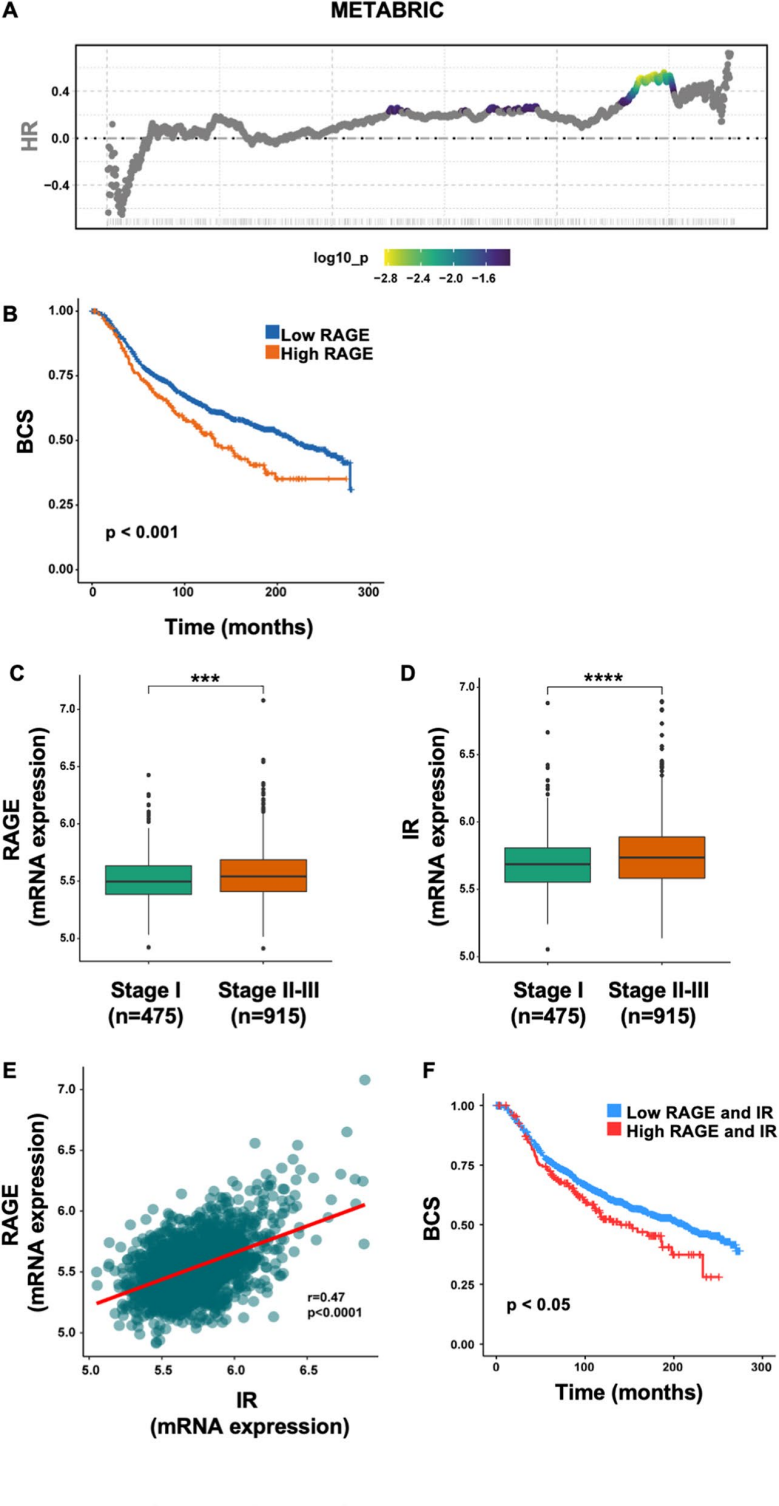
Co-expression of RAGE and IR in BC patients. Breast cancer patients of the METABRIC cohort [17] ordered according to RAGE expression levels (x-axis). The color bar gradient stands for range of the most significant points‐ of‐ separation of the population (low‐ high significance = blue‐ yellow gradient) based on RAGE expression and survival of each patient. **A** Kaplan–Meier curve showing the correlation between RAGE expression and breast cancer specific survival (BCS) in the METABRIC cohort of breast cancer patients (**B**). Box plot showing the expression levels of RAGE (**C**) and IR **D** in BC patients stratified by tumor stage. Scatter plot depicting the correlation between RAGE and IR expression levels in BC samples of the METABRIC cohort (**E**). The Pearson correlation coefficient (*r*) and the relative p-value are shown (**E**). Kaplan–Meier curve showing the correlation between RAGE expression and BCS in the METABRIC cohort of BC patients (**F**). (***) *p* < 0.001; (****) *p* < 0.0001

Previous studies have demonstrated that the functional cooperation between RAGE and IGF-1R drives the acquisition of malignant features in BC [16]. On the basis of these observations, we asked whether RAGE may also be implicated in the oncogenic actions mediated by the Insulin Receptor (IR), another relevant player of the IIGFs implicated in BC progression [6]. To this aim, we performed a bioinformatic analysis of METABRIC, a publicly available molecular dataset that collects gene expression and clinical data from a large cohort (*n* = 1904) of BC patients [17]. First, we found that RAGE is associated with worse BC-specific survival (Fig. 1A, B) and worse clinicopathological features in BC patients, as evidenced by a higher gene expression of RAGE in patients affected by stage II-III vs stage I BC (Fig. 1C). A similar trend was observed for IR, which was found more strongly expressed in BC patients affected by a more advanced stage of breast disease (Fig. 1D). Next, we determined that RAGE expression significantly correlates with the expression of IR in the METABRIC cohort (Fig. 1E). Such positive correlation was evidenced in both ER-positive and ER-negative BC subgroups (Additional file S1: Fig. S1 A-B). Interestingly, BC-specific survival in patients expressing simultaneously high levels of both RAGE and IR is lower compared with patients expressing low levels of both receptors (Fig. 1F). These observations suggest that RAGE and IR may cooperate toward the acquisition of aggressive features in BC patients.

### RAGE and IR cooperate in activating stimulatory transduction pathways

**Fig. 2:**
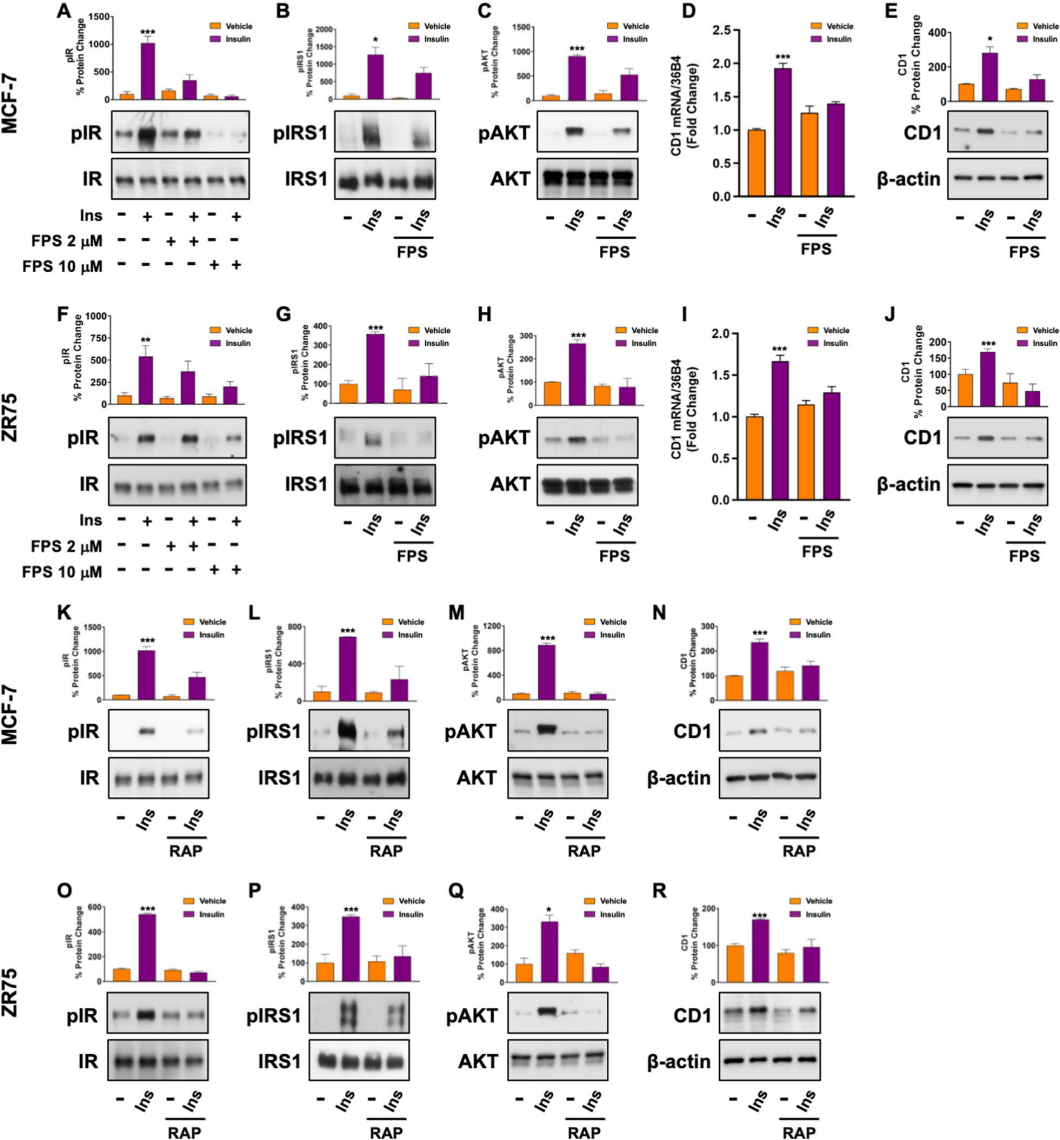
RAGE inhibition hampers Ins-induced signaling in BC cells. Representative immunoblots showing the phosphorylation of IR (Y1135/1136) (**A**, **F**, **K**, **O**), IRS1 (Y612) (**B**, **G**, **L**, **P**) and AKT (S473) **C**, **H**, **M**, **Q** in MCF-7 and ZR75 cells treated with Ins (20 nM, 15 min) alone and in combination with FPS-ZM1 (2 μM and 10 μM, 24 h) or RAP (50 μM, 24 h), as indicated. FPS-ZM1 (10 μM, 24 h) and RAP (50 μM, 24 h) interfere with the upregulation of CD1 mRNA (**D**, **I**) and protein expression **E**, **J**, **N**, **R** in MCF-7 and ZR75 cells stimulated with Ins (20 nM, 4 h), as indicated. Total proteins and β-actin serve as loading control. In qRT-PCR experiments, values are normalized to the 36B4 gene expression and shown as fold change of CD1 mRNA expression induced by Ins compared to cells treated with vehicle (−). Data shown are mean ± SEM of at least three independent experiments performed in duplicate. (*) *p* < 0.05; (***) *p* < 0.001

To get further insight into the potential of RAGE to facilitate BC progression in Ins-rich milieu, we used the estrogen receptor (ER-positive) BC cell lines MCF-7 and ZR75, which recapitulate the molecular features of the most frequently diagnosed BC histotype (Additional file S2: Fig. S2 A). First, we determined that in our experimental model Ins triggers the typical time-dependent activation of IR, IRS1 and AKT, as well as the up-regulation of the cell-cycle regulator cyclin D1 (CD1) (Additional file S2: Fig. S2 B-E). Next, we found that the transduction cascade IR/IRS1/AKT mediates the downstream up-regulation of CD1 (Additional file S2: Fig. S2 2 F-J. Then, we stimulated both MCF-7 and ZR75 BC cells with Ins, alone and in combination with increasing concentrations of the small molecule RAGE inhibitor FPS-ZM1 (2 μM ÷ 10 μM) (concentrations that did not interfere with cell viability as shown in Additional file S2: Fig. S2 K-L) [24], to evaluate whether the inhibition of RAGE signaling may affect Ins-induced stimulatory responses. Interestingly, 10 μM FPS-ZM1 were sufficient to hamper the activation of the IR/IRS1/AKT axis (Fig. 2A, C, Fig. 2F–H) induced by Ins in both cell models. In addition, FPS-ZM1 prevented the Ins-induced up-regulation of CD1 at both the mRNA and protein levels, as observed in MCF-7 and ZR75 BC cells subjected to qRT-PCR and Western blotting experiments (Fig. 2D, E, Fig. 2I, J).

Next, we confirmed the ability of RAGE to interfere with Ins-induced stimulatory pathways by using another pharmacological inhibitor, structurally unrelated to FPS-ZM1, named RAP (RAGE antagonist peptide), which represents an S100P-derived antagonist of RAGE [25]. Interestingly, we found that in both our experimental models RAP mitigates the activation of the IR/IRS1/AKT/CD1 pathway induced by Ins (Fig. 2K–R).

**Fig. 3:**
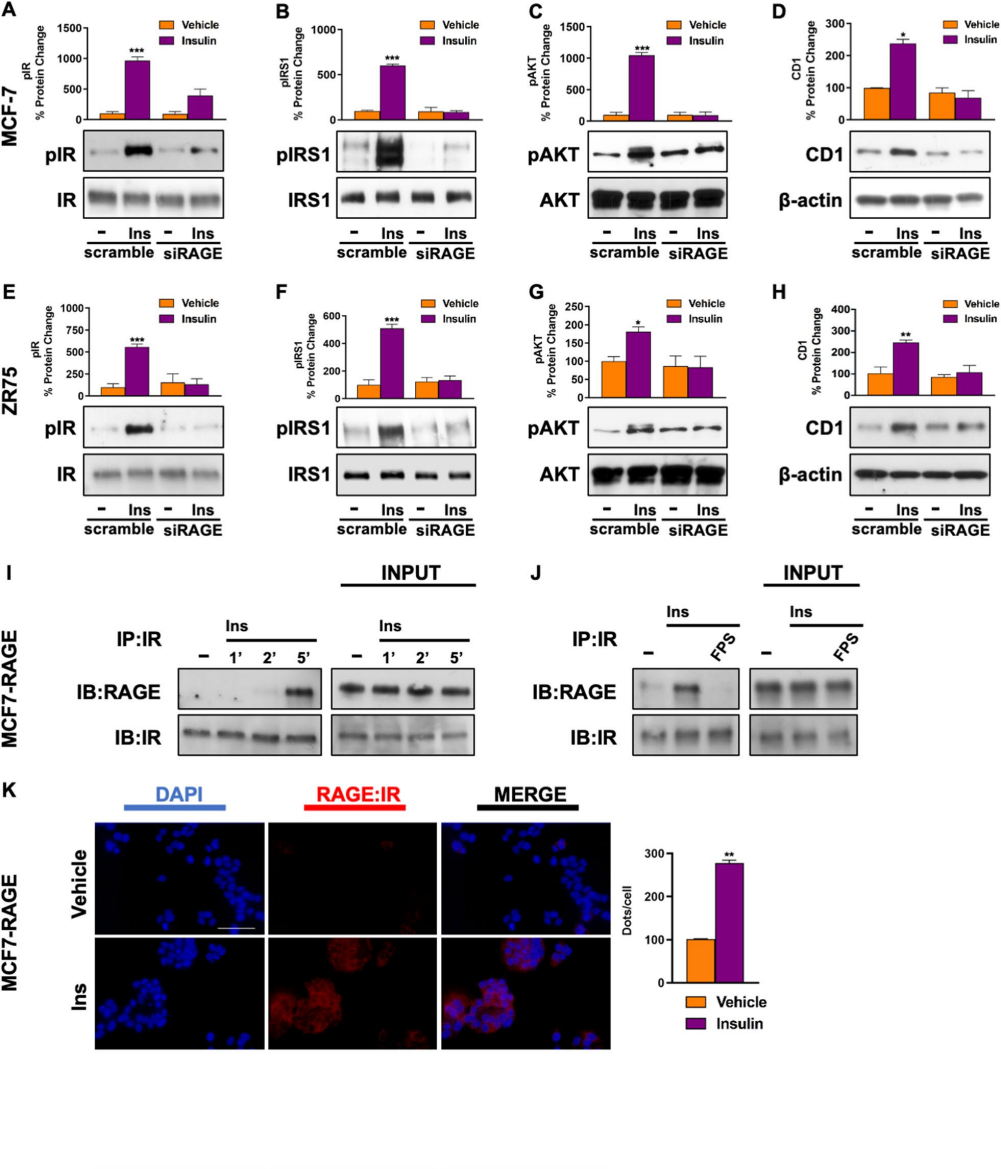
RAGE is involved in the activation of Ins/IR signals. The activation of IR (Y1135/1136), IRS1 (Y612) and AKT (S473) observed in MCF-7 (**A**–**C**) and ZR75 (**E**–**G**) cells treated with vehicle (−) or Ins (20 nM, 15 min) is abrogated by silencing RAGE. The up-regulation of CD1 observed in MCF-7 (**D**) and ZR75 **H** cells treated with vehicle (−) or Ins (20 nM, 4 h) is abrogated by silencing RAGE. Total proteins and β-actin serve as loading control. Co-immunoprecipitation of IR with RAGE in MCF-7 cells overexpressing RAGE (MCF7-RAGE) treated with Ins, as indicated (**I**). FPS-ZM1 (10 μM, 24 h) prevents the co-immunoprecipitation of IR with RAGE in cells stimulated with Ins (20 nM, 5 min) (**J**). Total lysates (input) are evaluated as control. In situ Proximity ligation assay in MCF7-RAGE cells treated with vehicle (−) or Ins (20 nM, 5 min). Red fluorescence indicates the membrane proximity of IR and RAGE (< 30–40 nm). Nuclei are stained by DAPI (blue fluorescence) (**K**). Side bar graph indicates the number of dots/cell. Data shown are the mean ± SEM of at least two independent experiments. (*) *p* < 0.05; (**) *p* < 0.01; (***) *p* < 0.001

Having established that the pharmacological inhibition of RAGE hampers Ins-mediated signaling in BC cells, we used a gene silencing approach to confirm our findings. As shown in Fig. 3, the activation of IR, IRS1 and AKT, as well as the up-regulation of CD1 protein expression induced by Ins in both MCF-7 (A-D) and ZR75 (E–H) cells was abolished in the presence of RAGE silencing (Additional file S2: Fig. S2 M-P).

To get further insight into the potential mechanism through which RAGE facilitates the stimulatory signals of Ins, we first generated an isogenic MCF-7 cell line overexpressing RAGE by means of lentiviral transduction. The efficiency of RAGE overexpression was confirmed by qRT-PCR and Western blotting (Additional file S3: Fig. S3 A-B). Western blotting experiments performed in the newly generated cell line allowed to establish that RAGE overexpression is sufficient to trigger the activation of IR signaling (Additional file S3: Fig. S3 C-D), as well as the up-regulation of CD1 (Additional file S3: Fig. S3 E), an effect that was abrogated by FPS-ZM1 (Additional file S3: Fig. S3 F). Next, co-IP assays revealed the direct association between RAGE and IR upon exposure to Ins for 5 min (Fig. 3I), while this effect was no longer evident in the presence of the RAGE inhibitor FPS-ZM1 (Fig. 3 J). RAGE and IR physical association upon Ins treatment was confirmed by performing in situ Proximity Ligation Assays (PLA), which detect protein-to-protein interaction through the evaluation of a fluorescent signal emitted only when two molecules exhibit a physical proximity lower than 30–40 nm (Fig. 3K and Additional file S3: Fig. S3 G). These results indicate that RAGE may act as a facilitator of Ins-initiated signals in BC cells by means of receptor–receptor interaction.

Previous studies have shown that the generation of a mild oxidative stress is necessary to prompt IR phosphorylation few minutes after cell stimulation with Ins [26]; in fact, it has been shown that the ROS scavenger N-acetyl-cysteine (NAC) interferes with Ins-induced phosphorylation of IR [27]. As RAGE activation is known to trigger ROS generation toward signal transduction [14], we asked whether the inhibition or the genetic depletion of RAGE may potentiate the effect of NAC on IR activation by Ins. In accordance with previous studies [27], we found that the addition of NAC interfered with IR phosphorylation in Ins-stimulated cells (Additional file S3: Fig. S3 H); furthermore, the addition of FPS-ZM1 as well as the silencing of RAGE potentiated the effects induced by NAC on IR activation upon Ins treatment (Additional file S3: Fig. S3 H-J). Collectively, these data suggest that ROS may be implicated in the cooperative cross-talk between RAGE and IR in Ins-treated BC cells.

### RAGE inhibition halts IR and IGF-1R-mediated signals

**Fig. 4:**
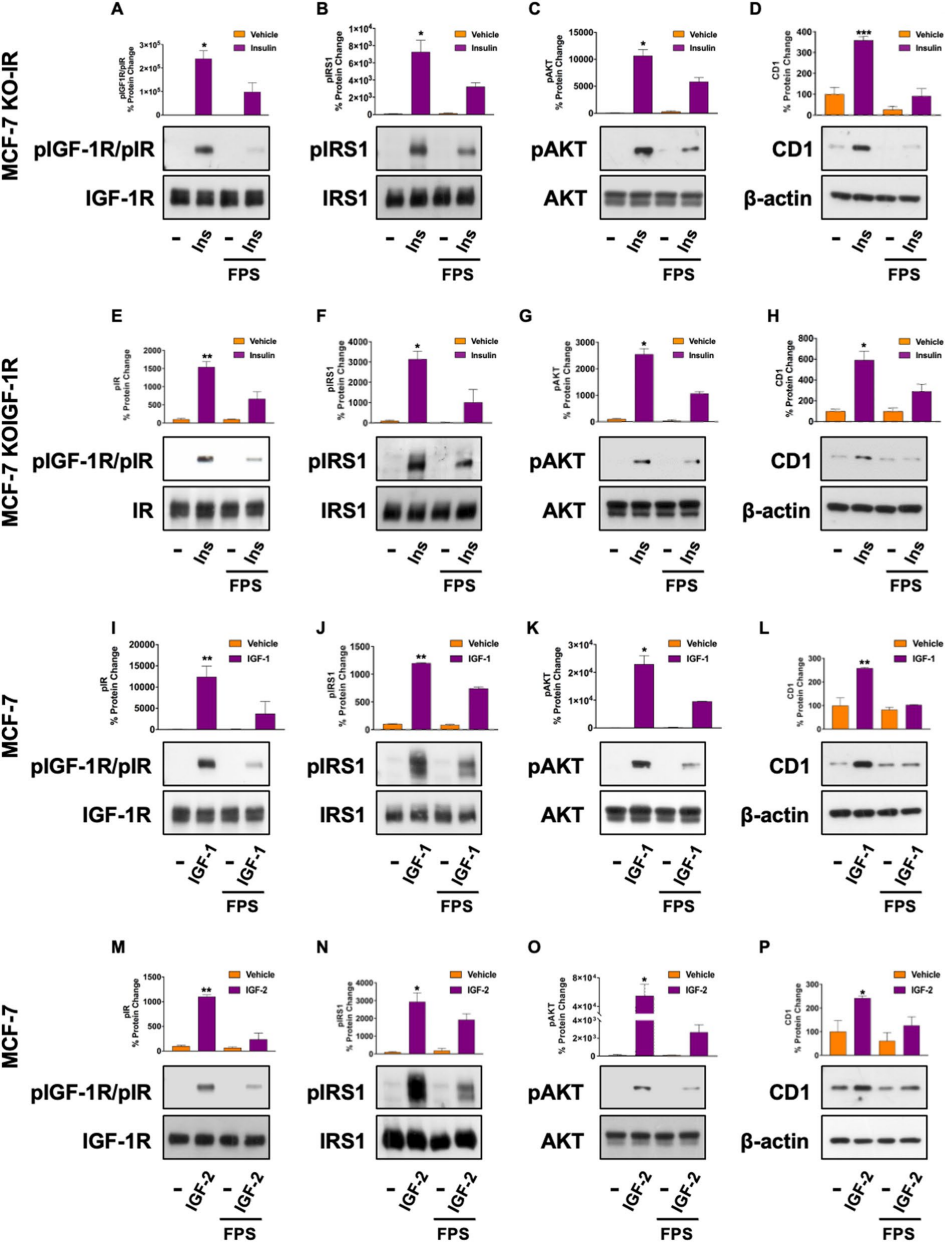
FPS-ZM1 halts IR and IGF-1R-mediated signals. The Ins-induced activation of IR/IGF-1R (Y1135/1136) (**A**), IRS1 (Y612) (**B**) and AKT (S473) (**C**), as well as the up-regulation of CD1 **D** is attenuated by FPS-ZM1 in MCF-7 cells engineered for the deletion of IR (KO-IR) (**E**). FPS-ZM1 interferes with the Ins-induced activation of IR/IGF-1R (Y1135/1136) (**F**), IRS1 (Y612) (**G**) and AKT (S473) (**H**), as well as the up-regulation of CD1 **I** in MCF-7 cells engineered for the deletion of IGF-1R (KOIGF-1R) Representative immunoblots showing the protein expression of pIR/pIGF-1R (Y1135/1136), pIRS1 (Y612), pAKT (S473) in MCF-7 cells treated for 15 min with IGF-1 (10 nM) (**I**−**K**) or IGF-2 (10 nM) (**M**, **O**), alone and in combination with FPS-ZM1 (10 μM, 24 h). Representative immunoblots showing the protein expression of CD1 in MCF-7 cells treated for 4 h with IGF-1 (10 nM) (L) or IGF-2 (10 nM) (**P**), alone and in combination with FPS-ZM1 (10 μM, 24 h). Total proteins and β-actin serve as loading control. Data shown are the mean ± SEM of at least three independent experiments. (*) *p* < 0.05; (**) *p* < 0.01; (***) *p* < 0.001

The Ins/IGF system (IIGFs) is a highly promiscuous transduction pathway; in fact, ligands belonging to IIGFs (namely Ins, IGF-1 and IGF-2) can signal through more than one receptor, despite with different affinities [5, 9]. For instance, Ins activates not only IR but also IGF-1R in diverse contexts including BC cells, further supporting the cooperation between IR and IGF-1R in the oncogenic actions mediated by IIGFs [5, 9]. On these bases, we set out to evaluate whether RAGE inhibition affects both IR and IGF-1R-mediated signals in Ins-rich milieu. To this aim, we first employed MCF-7 cells engineered for the deletion of the IR gene via CRISPR-cas9 genome editing. As shown in Fig. 4, Ins was still able to activate the IGF-1R/IRS1/AKT cascade, as well as to up-regulate CD1 protein levels in this model system, while FPS-ZM1 mitigated this effect (Fig. 4A–D and Additional file S3: Fig. S3 K-L). In addition, in MCF-7 cells knocked out for the IGF-1R gene via CRISPR-cas9 technology, FPS-ZM1 similarly attenuated the activation of the IR/IRS1/AKT cascade, as well as the up-regulation of CD1 protein expression induced by Ins (Fig. 4E–H and Additional file S3: Fig. S3 M–N). These data, suggesting that RAGE inhibition halts the IR- and IGF-1R-mediated actions, prompted us to test whether the inhibition of RAGE could interfere with the stimulatory signals induced by both IGF-1 and IGF-2, which are known to activate IR and IGF-1R in diverse physio-pathological contexts, including BC cells [28]. Interestingly, FPS-ZM1 reduced the activation of IR, IRS1, AKT, as well as the up-regulation of CD1 induced by both IGF-1 (Fig. 4I–L) and IGF-2 (Fig. 4 M–P) in MCF-7 cells. Altogether, these findings indicate that the inhibition of RAGE is sufficient to hamper the signaling pathways activated by IR and IGF-1R.

### Proteomic mapping reveals proteins and pathways affected by RAGE inhibition in Insulin-stimulated cells

**Fig. 5:**
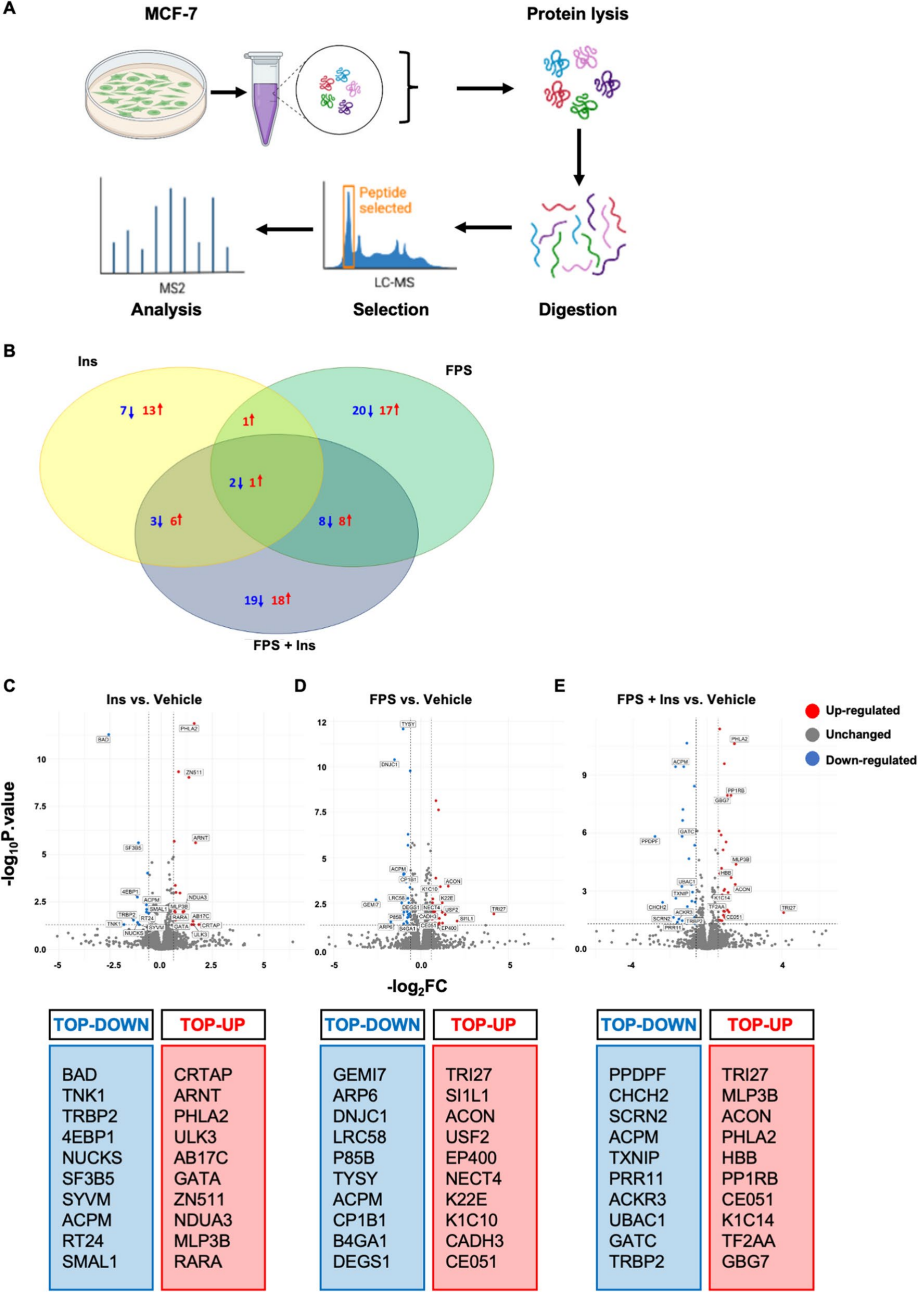
Proteomic analysis in MCF-7 cells. Schematic representation of experimental workflow for unbiased label-free proteomics study performed in MCF-7 cells exposed to Ins (20 nM, 4 h), alone and in combination with FPS-ZM1 (10 μM, 24 h) (**A**). Venn diagram showing the overlap of proteins differentially expressed (DEPs) across all treatments compared to vehicle-treated cells (B). Volcano plots showing the identified DEPs in cells stimulated with treatments compared to vehicle-treated cells, as indicated (**C**, **E**). X axis is the fold change (FC) (log2) of protein expression versus vehicle-treated cells, and the Y axis represents *p* (−log10). Red points (FC > 0.6) indicate up-regulated proteins; blue points (FC < 0.6) indicate down-regulated proteins, and gray points indicate proteins without significant differential expression. Bottom panels report the top-10 up-regulated (in red) and down-regulated (in blue) proteins in experimental groups

The results above suggest that inhibiting RAGE may halt Ins-initiated signals, which are known to control numerous transduction pathways and biological responses implicated in BC progression [8]. To depict the protein expression profile and predict signaling network landscape triggered by Ins and perturbed by RAGE inhibition, we used an “omics” approach. More specifically, unbiased label-free proteomics analysis was performed, according to a previously established protocol [21], in MCF-7 cells exposed to Ins, alone and in combination with FPS-ZM1. Briefly, whole cell protein lysates were subjected to digestion, peptide selection and analysis using LC–MS/MS [21] (Fig. 5A). Among proteins identified, we considered as differentially expressed all entries with an absolute Log2FC > 0.6 induced by ligands vs. vehicle-treated cells. Only proteins with a *p* value (*p*) ≤ 0.05 according to ANOVA were considered as differentially expressed. A heatmap of Differentially Expressed Proteins (DEPs) was generated (Additional file S4: Fig. S4). In all groups, we identified a total of 4656 proteins using Rstudio (R V 1.2.5033). Among DEPs, we found 96 up-regulated and 543 down-regulated in Ins-stimulated group compared to vehicle group; 89 proteins were up-regulated and 511 down-regulated in FPS-ZM1 group compared to vehicle group; 114 proteins were up-regulated and 591 down-regulated in FPS-ZM1 plus Ins group compared to vehicle group. Figure 5B shows the overlap of DEPs in experimental groups vs vehicle-treated samples. The top-10 DEPs in cells exposed to treatments vs. vehicle are schematically represented in Fig. 5C-E.

Among the top-10 up-regulated proteins in Ins-treated cells, we observed that the increase in NARFL (implicated in oxidative stress response) (NARFL: Log2FC = 3.48, *p* = 0.003) and ESF1 (implicated in ribosomal biogenesis) (ESF1: Log2FC = 2.82, *p* = 0.005) was significantly altered by FPS-ZM1 (NARFL: Log2FC = 3.19, *p* = 0.007), (ESF1: Log2FC = 2.6, *p* = 0.01). Furthermore, the Ins-induced up-regulation of UB2L6 (involved in ubiquitination) (UB2L6: Log2FC = 4.75, *p* = 0.01) and DHX40 (involved in RNA metabolism) (DHX40: Log2FC = 3.28, *p* = 0.04) was impaired by FPS-ZM1 (UB2L6: Log2FC = 2.79, *p* = 0.16), (DHX40: Log2FC = 2.15, *p* = 0.19). Interestingly, we found a significant increase in CD1 protein expression in MCF-7 cells stimulated with Ins (Log2FC = 0.8, *p* < 0.0001), whereas FPS-ZM1 interfered with this response (Log2FC = 0.6, *p* < 0.0001). Among down-regulated proteins, we found that the decrease in BAD (implicated in anti-apoptotic effects) (BAD: Log2FC = − 2.57, *p* < 0.05) and CDH2 (implicated in cell adhesion and dormancy) (CDH2: Log2FC = − 3.1, *p* = 0.002) induced by Ins was attenuated by FPS-ZM1 (BAD: Log2FC = − 1.3 with *p* < 0.05), (CDH2: Log2FC = − 1.5, *p* = 0.05). It should be mentioned that FPS-ZM1 triggered a significant additive effect only on one protein among the top-10 DEPs regulated by Ins: the up-regulation of TRI27 (Log2FC = 3.34 *p* = 0.02) induced by Ins was significantly potentiated by FPS-ZM1 (Log2FC = 4.11 and *p* = 0.0002).

**Fig. 6:**
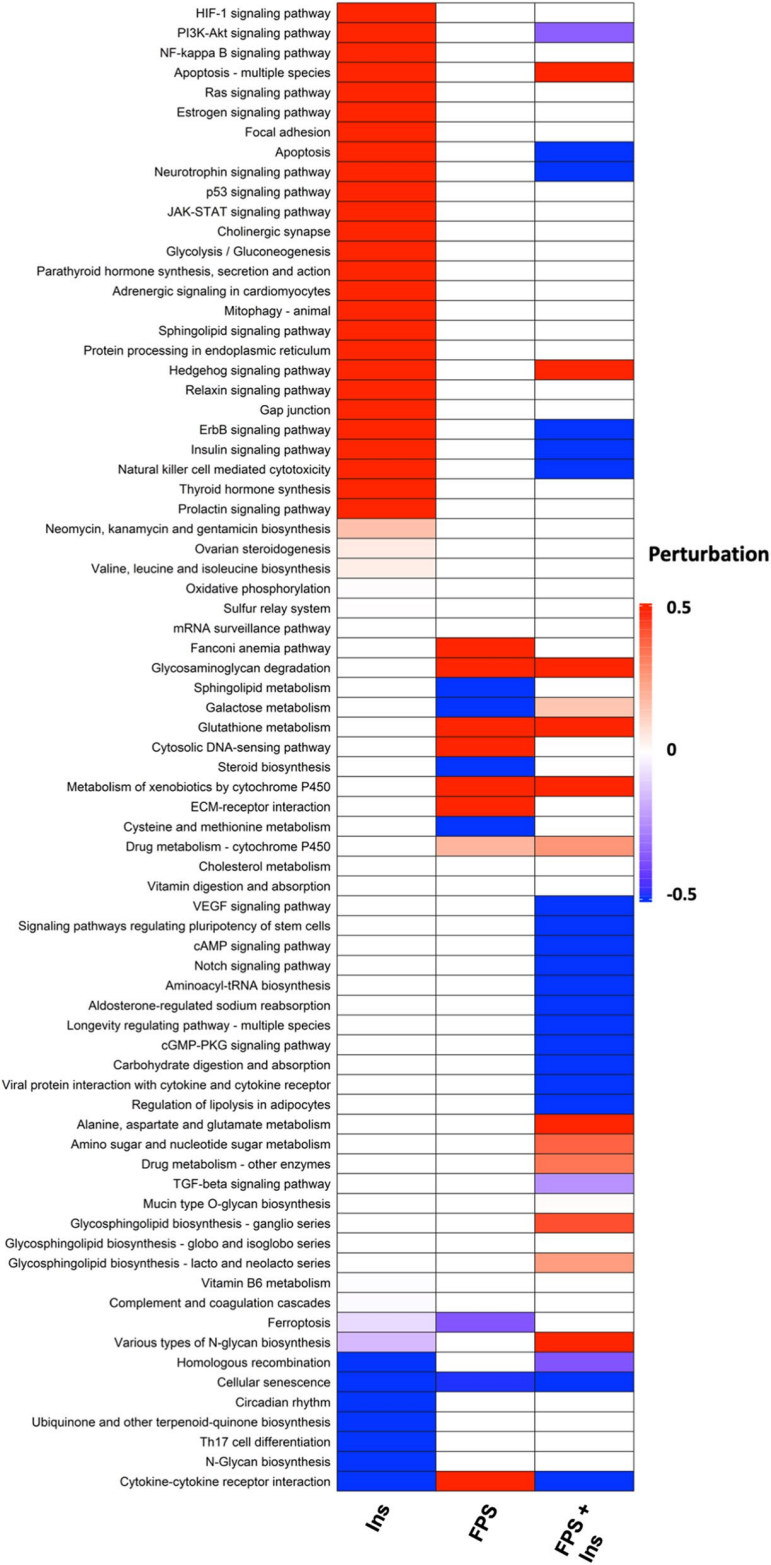
Analysis of the dysregulated pathways. Heatmap showing significantly dysregulated pathways, ordered by Ins, found by MITHrIL using the results of the proteomic data analysis. Pathways were colored accordingly to their corrected accumulator values calculated by the MITHrIL algorithm. The red color means upregulation, while the blue color means downregulation, as indicated. Pathways that were not found to be statistically dysregulated are colored in white

Next, we used the algorithm MITHrIL [29] to perform pathway analysis on identified DEPs. The underlying pathway topologies were obtained from Kyoto Encyclopedia of Genes and Genomes (KEGG) database [30]. Top-50 perturbated pathways ordered by Ins are shown in Fig. 6.

According to MITHrIL results, certain signaling pathways were predicted to be significantly up-regulated in Ins-treated cells, including: (1) pathways involved in hormones and growth factor signaling (Insulin signaling, Estrogen signaling, ErbB signaling, Thyroid hormone synthesis, Parathyroid hormone synthesis, Prolactin signaling pathway, Ovarian steroidogenesis); (2) pathways involved in the transduction of proliferative, adhesion and pluripotency signals (PI3K-Akt signaling, Ras signaling, JAK/STAT signaling, Hedgehog signaling, Focal adhesion pathway); (3) pathways involved in cell response to stress and inflammation (HIF-1 pathway, NF-kappa B signaling); (4) pathways implicated in cell metabolism (glycolysis/gluconeogenesis, mitophagy pathway, valine, leucine and isoleucine biosynthesis); (5) pathways involved in immune regulation (natural killer cell mediated cytotoxicity) (Fig. 6). Interestingly, in cells stimulated with Ins, the addition of FPS-ZM1 interfered with the abovementioned pathway perturbations, which were found to be less up-regulated, down-regulated or not significantly perturbated (Fig. 6, Additional file 8: Table S1). Finally, other signaling pathways were found to be down-regulated upon Ins stimulation, including: (1) ferroptosis; (2) N-glycan biosynthesis; (3) homologous recombination; (4) cellular senescence; (5) ubiquinone and other terpenoid-quinone biosynthesis; (6) cytokine-cytokine receptor interaction; (7) Th17 cell differentiation (Fig. 6). Of note, in cells stimulated with Ins, the addition of FPS-ZM1 interfered with the abovementioned perturbation of pathways, which were found to be less down-regulated, up-regulated or not significantly perturbated (Fig. 6, Additional file 8: Table S1).

A topological representation of MITHrIL outputs for the PI3K/AKT pathway, which is a crucial translational effector of Ins-initiated signals, is shown in Additional file S5: Fig. S5. Interestingly, nodes associated with cell proliferation, survival and antiapoptotic effects appeared to be upregulated by Ins, whereas FPS-ZM1 attenuated this effect.

Altogether, these data expose, from an omics perspective, the signaling routes perturbated by RAGE inhibition upon Ins stimulation.

### IR and RAGE cooperate in activating biological responses involved in BC progression

**Fig. 7:**
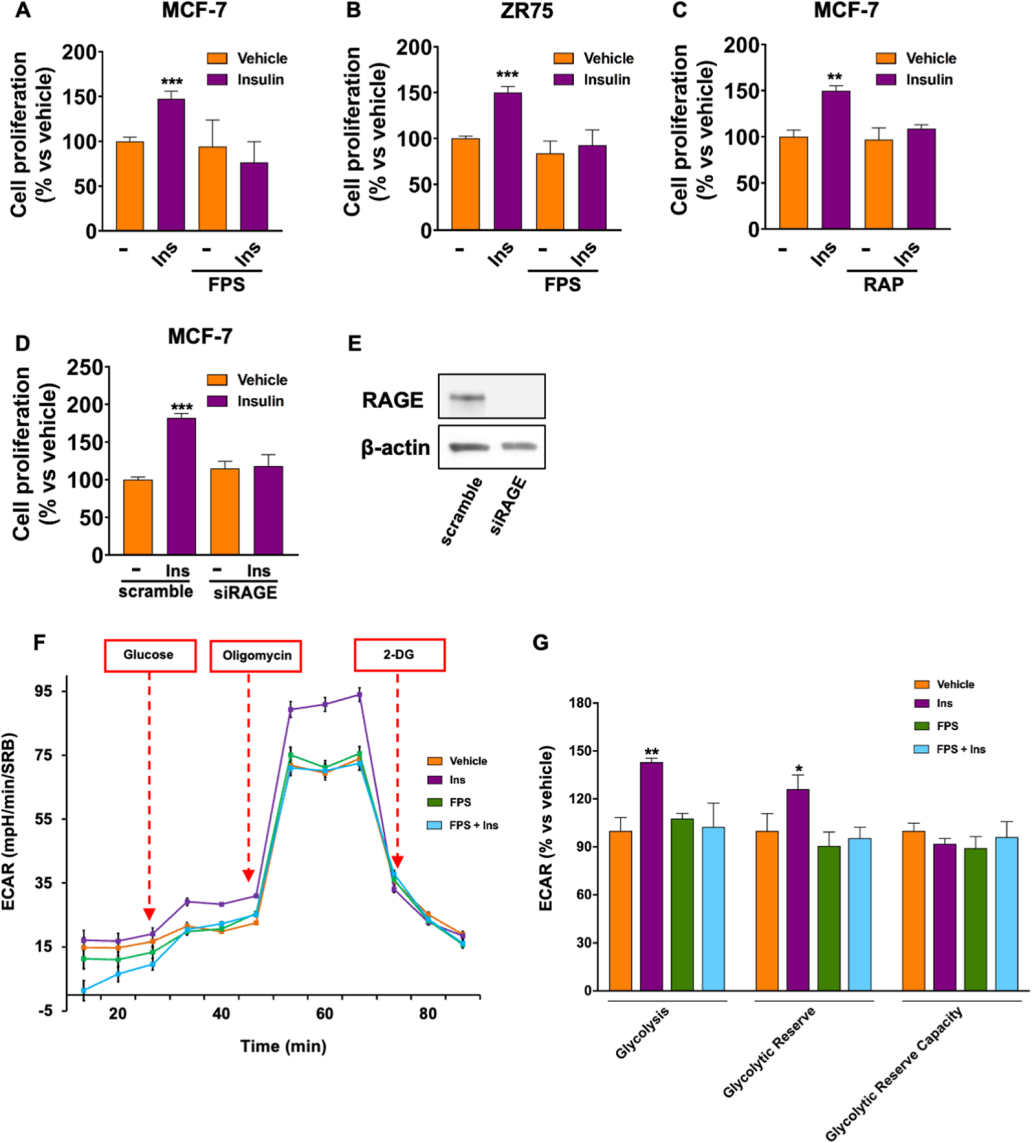
RAGE inhibition prevents Ins-induced cell proliferation and glucose utilization in MCF-7 cells. Evaluation of cell proliferation by cell counting in MCF-7 (**A**) and ZR75 **B** cells treated with Ins (20 nM, 72 h), alone and in combination with FPS-ZM1 (10 μM), or RAP (50 μM) (**C**). Evaluation of cell proliferation in MCF-7 cells transfected with siRAGE (10 nM) or non-targeting scramble siRNA for 24 h before stimulation with Ins (20 nM, 72 h) (**D**). Efficacy of RAGE silencing as evaluated by Western blotting (**E**). Representative tracing of Extracellular acidification rate (ECAR) in MCF-7 cells treated with Ins (20 nM, 8 h) alone and in combination with FPS-ZM1 (10 μM, 24 h) (**F**). Evaluation of glycolysis, glycolytic reserve and glycolytic reserve capacity (**G**). Data shown are the mean ± SEM of at least two independent experiments. (*) *p* < 0.05; (**) *p* < 0.01; (***) *p* < 0.001

Having established that RAGE cooperates with IR in mediating Ins-initiated signaling in BC cells, we aimed to evaluate whether RAGE inhibition may interfere with the biological responses prompted by Ins. Of note, the increase in cell proliferation observed in Ins-stimulated MCF-7 and ZR75 cells was abrogated in the presence of the RAGE inhibitors FPS-ZM1 and RAP (Fig. 7A, C), as well as depleting RAGE levels by gene silencing (Fig. 7D, E). Consistent with the instigation of a quiescent cell phenotype, FPS-ZM1 abrogated the Ins-induced activation of the glycolytic flux, as evaluated by Seahorse analysis (Fig. 7F, G), whereas Ins did not determine significant changes in OXPHOS metabolism in the same experimental conditions (Additional file S3: Fig. S3 O).

**Fig. 8:**
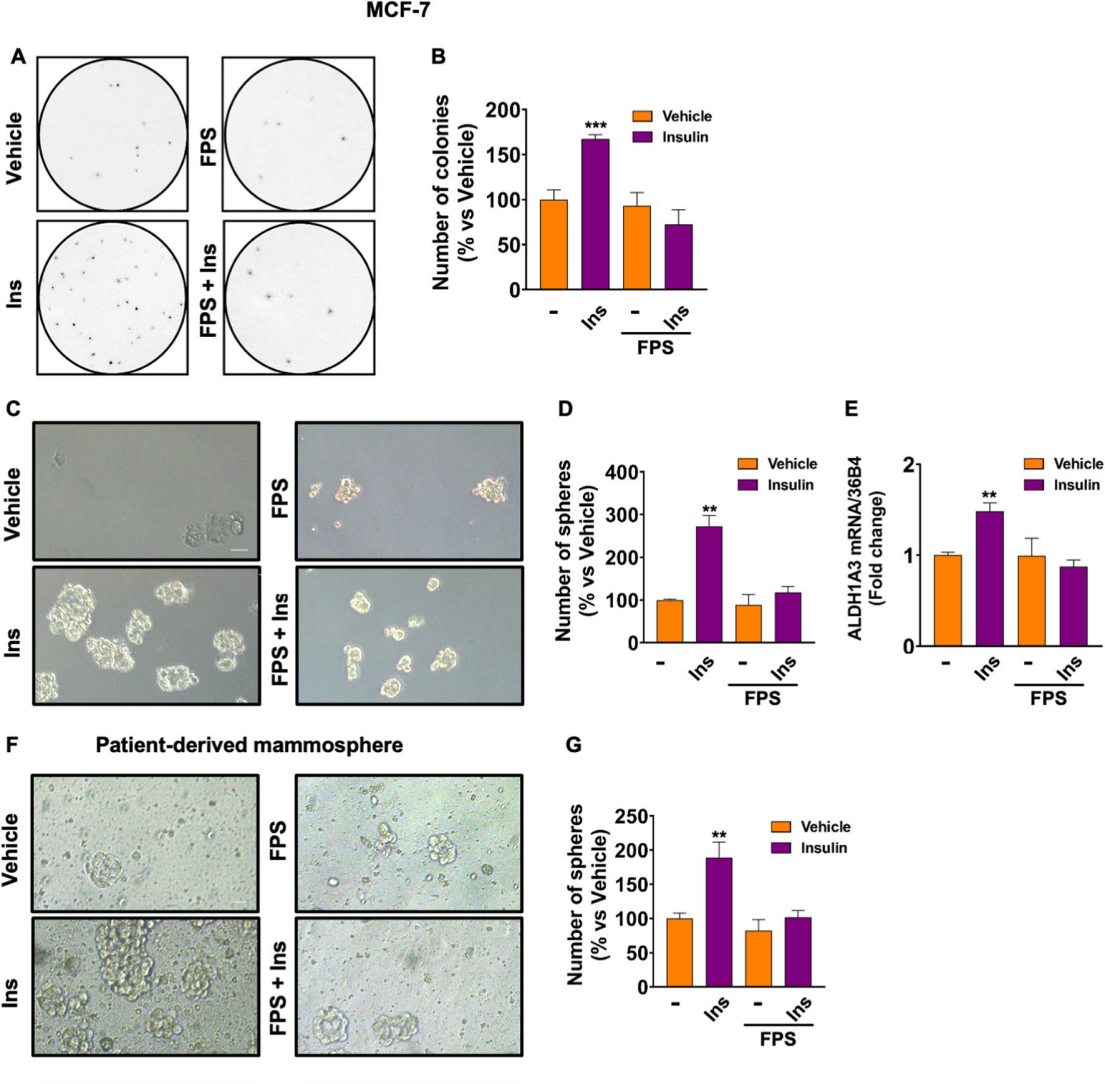
RAGE inhibition prevents colony and mammosphere formation induced by Ins. Evaluation of colony formation in MCF-7 cells treated with Ins (20 nM, 3 weeks), alone and in combination with FPS-ZM1 (10 μM) (**A**, **B**). Representative images showing mammosphere formation in MCF-7 cells treated with Ins (20 nM), alone and in combination with FPS-ZM1 (10 μM) for 5 days. Only spheres with a diameter > 50 μm were counted. Scale bar: 50 μm (**C**, **D**). mRNA expression of ALDH1A3 in MCF-7 cells treated with vehicle (−) or Ins (20 nM, 4 h) alone and in combination with FPS-ZM1 (10 μM, 8 h), as evaluated by qRT-PCR. Values are normalized to the 36B4 gene expression and shown as fold changes of mRNA expression induced by Ins compared to cells treated with vehicle (**E**). Evaluation of mammosphere formation in cells derived from surgical specimens obtained from BC patients. Cells were isolated and grown in suspension culture in the presence of Ins (10 nM), alone and in combination with FPS-ZM1 (10 μM) for 10 days (**F**). Only spheres with a diameter > 50 μm were counted (**G**). Data shown are the mean ± SEM of at least two independent experiments. (**) *p* < 0.01; (***) *p* < 0.001

Further extending these findings, FPS-ZM1 abrogated colony formation in MCF-7 cells cultivated in soft-agar and stimulated with Ins (Fig. 8A, B). These data suggest that RAGE inhibition interferes with Ins-stimulated anchorage-independent growth, which is a relevant feature of cancer stem cells (CSCs). Hence, we performed mammosphere formation assays as a read-out for CSC activity. Of note, we found that the number of mammospheres increases in MCF-7 cells cultivated in low attachment conditions and stimulated with Ins compared with vehicle-treated cells (Fig. 8C, D); however, this effect was no longer evident in the presence of FPS-ZM1 (Fig. 8C, D). Likewise, the up-regulation of the well-acknowledged stemness marker ALDH1A3 induced by Ins was prevented in the presence of FPS-ZM1 (Fig. 8E). Next, we confirmed these findings using patient-derived samples and employing an *ex vivo* approach characterized by higher translational predictivity compared with in vitro strategies. To this aim, bioptic fragments deriving from BC patients were digested and grown in low attachment conditions as single cell suspensions for 10 days in the presence of Ins, alone and in combination with FPS-ZM1. Relevant clinical information for each patient is reported in Additional file 9: Table S2. Interestingly, RAGE inhibition halted Ins-induced mammosphere formation in patient-derived BC cells (Fig. 8F, G).

**Fig. 9:**
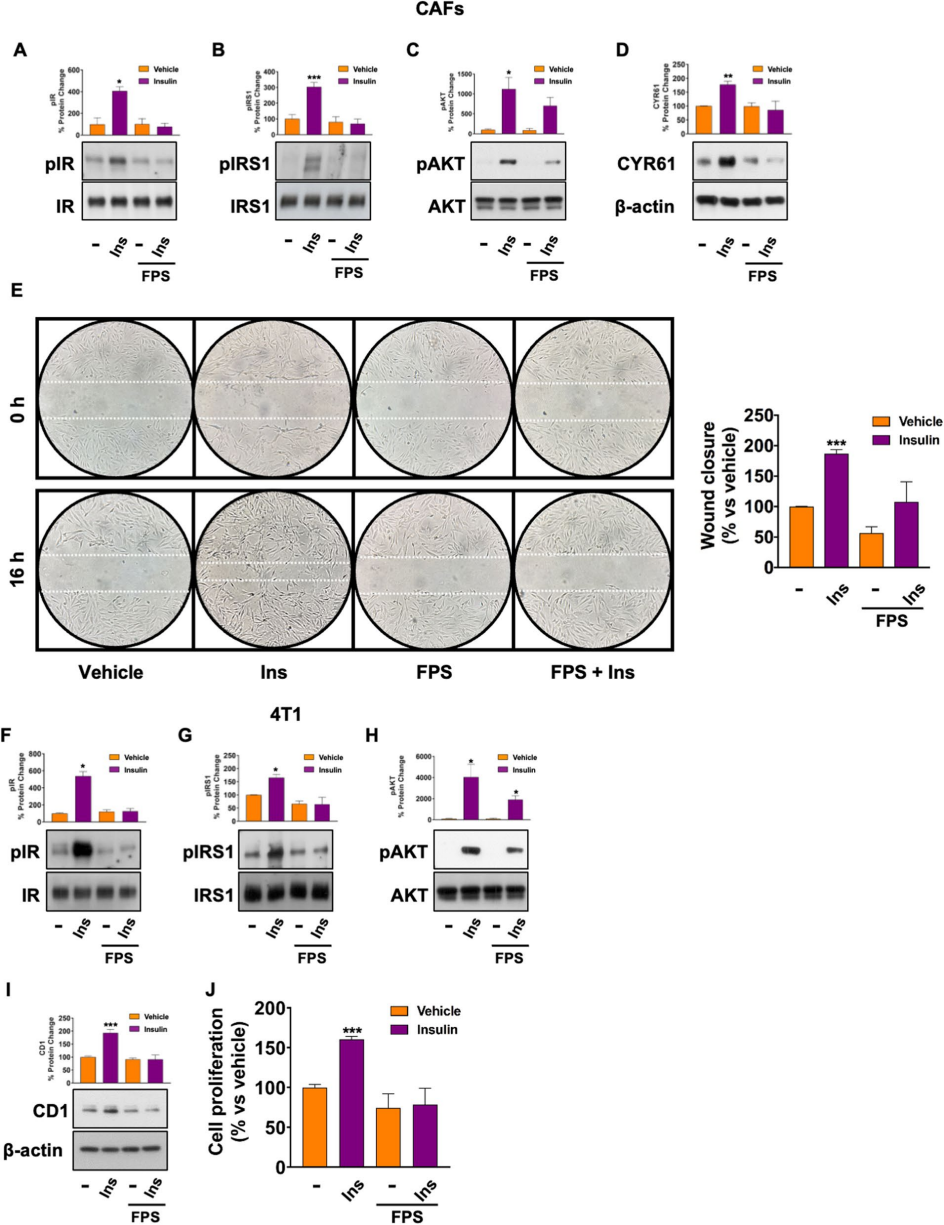
FPS-ZM1 interferes with Ins-induced stimulatory pathways in CAFs and 4T1 cells. Representative immunoblots showing the phosphorylation of IR (Y1135/1136) (**A**), IRS1 (Y612) (**B**), and AKT (S473) (**C**) in CAFs treated with Ins (20 nM, 15 min) alone and in combination with FPS-ZM1 (10 μM, 24 h). Treatment with FPS-ZM1 (10 μM, 24 h) abrogates the up-regulation of CYR61 protein expression **D** in CAFs stimulated with Ins (20 nM, 4 h). Total proteins and β-actin serve as loading control. Wound healing assay in CAFs scratched and treated with Ins (20 nM) alone and in combination with FPS-ZM1 (10 μM) in serum free medium. Images are acquired at 0 and 16 h after scratching, as indicated (**E**). Side panel shows the quantification of cell migration expressed as % of wound closure. Representative immunoblots showing the phosphorylation of IR (Y1135/1136) (**F**), IRS1 (Y612) (**G**), and AKT (S473) (**H**) in 4T1 cells treated with Ins (20 nM, 15 min) alone and in combination with FPS-ZM1 (10 μM, 24 h). Treatment with FPS-ZM1 (10 μM, 24 h) abrogates the up-regulation of CD1 protein expression in 4T1 cells stimulated with Ins (20 nM, 4 h) (**I**). Total proteins and β-actin serve as loading control. 4T1 cells were seeded in 96-well plates and treated with either vehicle (−) or Ins (20 nM), alone and in combination with FPS-ZM1 (10 μM) for 72 h before evaluation of cell growth by SRB assay (**J**). Data shown are mean ± SEM of at least three independent experiments performed in duplicate. (*) *p* < 0.05; (**) *p* < 0.01; (***) *p* < 0.001

To further dissect the cross-talk between IR and RAGE in BC, we used bioptic fragments from BC patients to isolate Cancer-Associated Fibroblasts (CAFs), which represent a critical component of the tumor microenvironment involved in disease progression [31]. First, CAFs were isolated and characterized by immunofluorescence and RT-PCR analysis (Additional file S6: Fig. S6 A-B.). Interestingly, we found that also in this experimental model, FPS-ZM1 attenuates the activation of the IR/IRS1/AKT cascade induced by Ins (Fig. 9A–C). Conversely, we could not detect any increase in CD1 expression and cell proliferation in CAFs stimulated with Ins (data not shown). However, a marked up-regulation of the migratory protein CYR61 was observed in Ins-treated CAFs (Fig. 9D), whereas FPS-ZM1 abolished this effect (Fig. 9D). Accordingly, Ins-induced migration of CAFs was reversed by FPS-ZM1 (Fig. 9E).

### RAGE inhibition hampers mammary tumor growth induced by Insulin

**Fig. 10:**
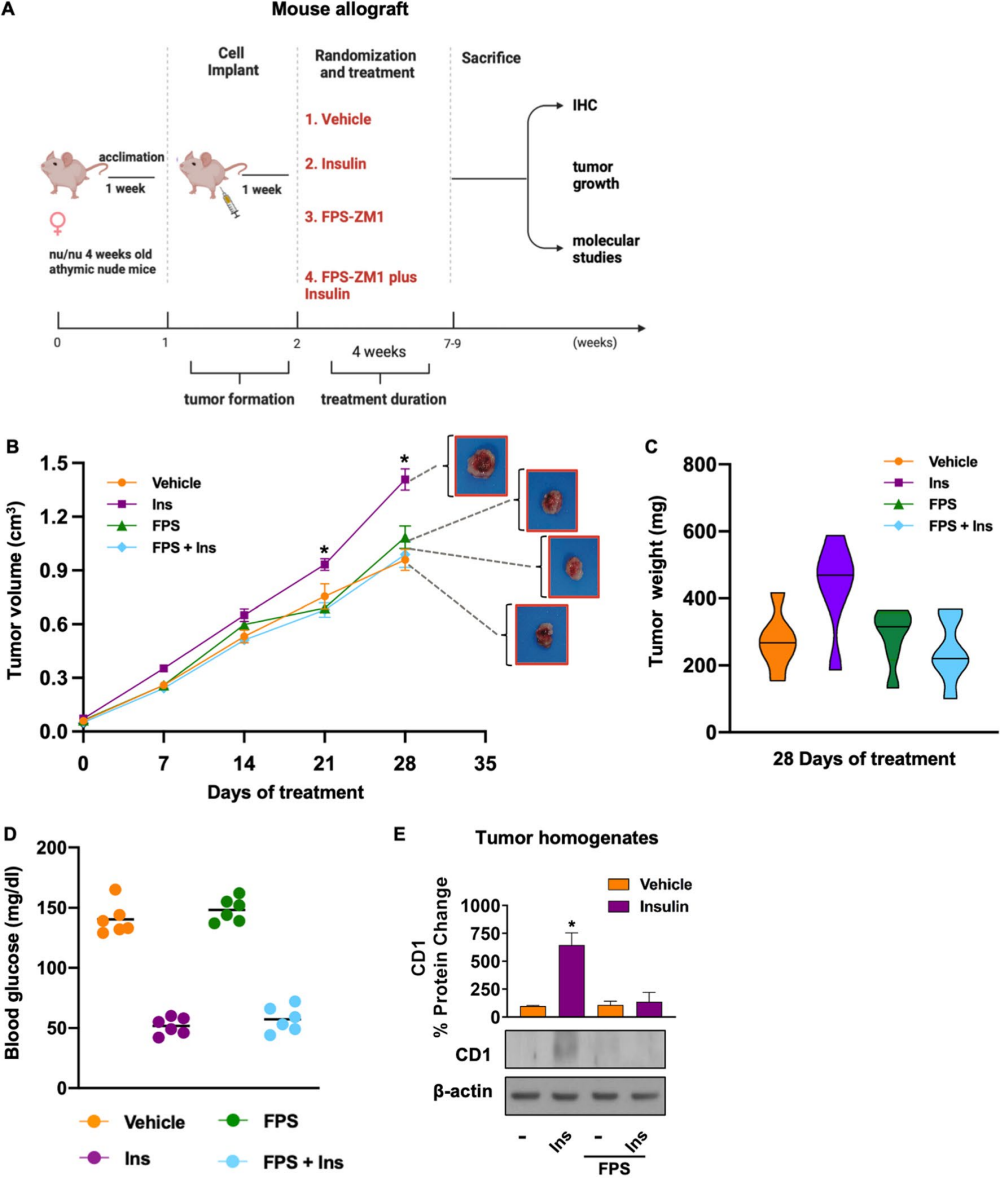
FPS-ZM1 interferes with Ins-induced growth of 4T1-derived allografts. Schematic representation of the experimental workflow for the in vivo studies. Female athymic nude mice were inoculated with 4T1 cells. On the seventh day after inoculation, mice were treated with 10 nM Ins glargine, given s.c. for 5 days/week, alone and in combination with FPS-ZM1 (1 mg/kg) given i.p., twice a week (*n* = 6 for each group). At day 28, mice were sacrificed and tumor tissue was collected (**A**). Graph showing the tumor volumes (cm3) at indicated time points from the starting of treatments. Representative images of explanted tumors are shown. The data are the mean ± SEM of the values obtained in six animals per group. (*) *p* < 0.05 (**B**). Tumors’ weight in mice at sacrifice (**C**). Evaluation of blood glucose levels in mice, as indicated (**D**). Representative immunoblot showing the evaluation of CD1 protein expression in tumor homogenates from 4T1-derived allografts. β-actin serves as loading control (**E**). Data shown are mean ± SEM of at least three independent experiments. (*) p < 0.05

To extend the data obtained in in vitro experimental systems, we turned to an in vivo allograft rodent model, which better recapitulates the features of the human breast tumor microenvironment, thus providing a useful tool for pre-clinical validation in drug screening and discovery. To this aim, we employed the highly tumorigenic murine mammary 4T1 BC cell line that we previously used successfully in animal studies aimed at dissecting the role of IR in BC progression [6]. We first assessed that this cell model expresses RAGE and IR (Additional file S2: Fig. S2 A); next, we determined that also in 4T1 cells, RAGE inhibition by FPS-ZM1 mitigates the activation of the IR/IRS1/AKT/CD1 cascade (Fig. 9F–I), as well as the proliferative effects (Fig. 9J) induced by Ins. Thereafter, 4T1 tumor cell allografts were performed by injecting cells into the mammary fat pad regions of 4-weeks-old female athymic nude mice. Subsequently, mice were treated vehicle, Ins Glargine alone and in combination with FPS-ZM1 (Fig. 10A). These administrations were well tolerated, as evidenced by the lack of alterations in mice body weight, food and water consumption and motor function. In addition, no significant difference in the mean weights or histologic features of the major organs (liver, lung, spleen, and kidney) was observed after sacrifice in all experimental groups, thus indicating a lack of toxic effects. Tumor growth was monitored twice a week. Starting from day 21 and up to day 28 of treatment, FPS-ZM1 was able to halt Ins-induced tumor growth (Fig. 10B, C), without affecting blood glucose levels (Fig. 10D). Representative tumor images and morphologic analyses of histological 4T1 allograft tumor sections are shown in Fig. 10B and Additional file S6: Fig. S6 C, respectively.

Next, we found that the protein expression of the cell cycle regulator CD1 increases in tumor homogenates (Fig. 10E) and tumor tissues slides (Additional file S6: Fig. S6 D) obtained from Ins-stimulated mice compared with vehicle-treated mice; however, these stimulatory effects were abrogated in the animal group receiving FPS-ZM1 in addition to Ins (Fig. 10E and Additional file S6: Fig. S6 D). Altogether, these observations suggest that RAGE inhibition halts BC growth induced by Ins in vivo.

## Discussion

In the present study, we investigated the functional cooperation between IR and RAGE toward the activation of stimulatory responses in BC. We established that IR and RAGE are co-expressed in BC patients and correlate with worse clinical parameters and prognostic outcomes. By using cell models that recapitulate the features of both ER-positive and ER-negative breast malignancies, we assessed that the pharmacological inhibition of RAGE, as well as its genetic depletion, interferes with the activation of the oncogenic pathway IRS1/AKT/CD1 induced by Ins. Mechanistically, we showed that Ins prompts an early direct interaction between IR and RAGE, likely implicated in IR activation and signal transduction. We ascertained that RAGE contributes to the pro-tumorigenic responses triggered by Ins/IR in BC as demonstrated using in vitro, in vivo, *ex vivo* approaches and patient-derived samples. Therefore, the present findings provide novel insights into the potential of RAGE to facilitate Ins/IR signaling, thus serving as a promising pharmacological target in BC.

Epidemiological studies indicate a clear correlation between impaired metabolic health and the development and progression of diverse types of tumors, including BC [1–3]. For instance, obesity and type 2 diabetes are associated with increased risk of BC, higher metastatic propensity, refractoriness to conventional and targeted therapies, and poor prognosis [3, 32, 33]. Impaired Ins signaling and hyperinsulinemia are frequently observed in obese, prediabetic and diabetic individuals, suggesting that this hormone may play a key role in the pathogenesis and progression of BC during metabolic imbalances. Likewise, high circulating levels of Ins are associated with BC relapse, therapeutic resistance, as well as poor prognosis [34–37]. In this regard, the data we collected from animal studies, showing that Ins stimulates BC growth, provide a further solid rational for the well-known epidemiological link between hyperinsulinemia and BC progression and are backed up by previous investigations [38, 39].

To understand the root of hyperinsulinemia in dysmetabolic obese patients, the metabolic actions of this peptide hormone have to be considered. Ins serves as a master regulator of energy metabolism and global glucose flux balance [40]. By internalizing glucose within cells, Ins responds to glycemic spikes that occur after food intake, thus contributing to maintain sugar homeostasis [7]. The metabolic effects of Ins are mainly mediated by the Insulin Receptor (IR), a transmembrane RTK that enables glucose internalization within cells and suppresses hepatic gluconeogenesis, ultimately triggering blood glucose-lowering effects [41]. However, in obese and diabetic patients, the metabolic actions of Ins are impaired and the effectiveness of the Ins/IR pathway is compromised [14]. As a result, blood glucose levels may remain elevated and a compensatory increase in Ins production and release is enacted by Ins-producing cells [14]. The deriving hyperinsulinemia represents an important risk factor and negative prognostic indicator for the development and progression of neoplastic conditions like BC [14]. In fact, the activation of IR by Ins prompts BC cell proliferation, migration, invasion and angiogenesis in ER-positive and ER-negative models [6, 42, 43]. Likewise, the expression of IR is associated with lower overall survival in BC patients [6]. In accordance with these findings, our data indicate a higher expression of IR in BC patients affected by more advanced stages of breast tumor.

IR exists in two isoforms that derive from an alternate splicing of the IR gene [5]. Such an event, which represents an evolutionary conserved mechanism in mammals, generates the isoform A which lacks exon 11, thus resulting in a slightly shorter protein compared with the full-length isoform B. While IR-B is known to mediate the metabolic effects of Ins, IR-A appears to be implicated in non-metabolic effects such as embryonic growth and fetal development [5]; of note, in adult life IR-A has been involved in aging, hyperproliferative disorders and cancer [44]. In this context, our recent study has highlighted that IR-A-mediated effects play a crucial role in BC growth, angiogenesis and metastasis, as demonstrated using both in vitro and in vivo experimental approaches [6].

As an immediate outcome of these findings, therapies reducing Ins/IR-A signaling could attenuate the likelihood of malignant progression, particularly in obese BC patients. Nevertheless, pharmacological agents specifically directed at IR-A are not currently available; the lack of a drug able to discriminate and target IR-A exclusively, sparing IR-B mediated actions, explains the objective difficulties met when trying to control BC progression by using IR-targeting agents, as these strategies would precipitate a diabetic-like state in patients. To avoid these undesirable effects, certain anti-diabetic and Ins sensitizers like metformin have been investigated in drug-repurposing efforts with promising observational and pre-clinical results [43, 45]. However, the clinical translation of these findings has frustrated the great expectations [46]. Therefore, additional research efforts are required to better dissect Ins/IR signaling, regulatory networks and transduction companion, in order to identify novel and more effective targets of pharmacological intervention in BC. In light of these observations, our study indicates that RAGE serves as a previously unidentified modulator of Ins/IR signaling in BC and a promising pharmacological target, particularly in hyperinsulinemic and/or obese BC patients.

Being implicated in innate immunity and inflammation, RAGE and its ligands are largely recognized as pivotal contributors to the detrimental phenotypes observed in obese and diabetic subjects [14]. Indeed, the activation of RAGE in the adipose tissue contributes to the instigation of low-grade chronic inflammation, toward the establishment of Ins resistance and hyperinsulinemia [47]. The mechanisms involved include the RAGE-mediated regulation of ROS (reactive oxygen species), together with the NF-κB dependent inflammatory reprogramming [48–50]. Accordingly, genetic depletion of RAGE prevents weight gain, adipose tissue inflammation and impairment of Ins action in animal models of diet-induced obesity [51]. Beyond its implication in obesity and diabetes, RAGE is emerging as a pivotal orchestrator of tumor-promoting responses also in BC. In this regard, higher expression of RAGE was found in 25 BC specimens compared with non-cancerous tissues [52]; in addition, RAGE immunoreactivity correlated with advanced tumor stage, node positivity, tumor size [52] and lower overall survival [53]. Extending these findings, our bioinformatic analyses show that high expression levels of RAGE correlate with lower BC-specific survival and worse clinical features in a large cohort of human BC patients, totaling almost 2000 samples [17]. Interestingly, we assessed a positive correlation between RAGE and IR expression in the same cohort, supportive of a potential cross-talk between RAGE and IR in BC. Nicely fitting with these observations, patients expressing high levels of both RAGE and IR showed lower BC-specific survival, compared with patients expressing low levels of both receptors.

Our in vitro data support the functional cooperation between IR and RAGE in both ER-positive and Triple Negative BC cell models, which recapitulate the features of the most frequently diagnosed and the most therapeutically challenging types of BC, respectively [54]. We found that the pharmacological inhibition of RAGE is sufficient to halt the activation of oncogenic pathways and biological responses in Ins-rich environments. These findings add to the current knowledge in the field of IR signaling and associated molecular partners and indicate that a direct interaction between RAGE and IR contributes to conveying molecular signals and biological responses in BC cells. It should be mentioned that our Co-IP and PLA assays, showing RAGE and IR interaction upon Ins stimulation, have been performed in cell models engineered for the overexpression of RAGE; as overexpression may lead to protein mislocalization, future studies are warranted to evaluate RAGE/IR physical interaction in more physiologically-resembling experimental models, in order to better extend the repertoire of IR-interacting proteins. In this regard, previous studies have identified the non-integrin collagen RTK named discoidin domain receptor (DDR1) as an IR-interacting protein [55]. Not only DDR1 co-localizes with IR in response to the IR ligands Ins and IGF-2, but it also contributes to regulate IR expression, as well as ligand-dependent activation of signal transduction, energetic metabolism and biological responses in BC cells [55, 56]. Hence, the manipulation of DDR1 may be clinically relevant in those malignancies associated with dysregulated IIGFs. While the efficacy of DDR1 blocking strategies as a pharmacological mean to control disease progression has been successfully tested in in vitro studies [20], more controversial findings have been reported in animal models [57–59]. In addition, the potential anti-cancer effect of DDR1 ablation in animal models mimicking hyperinsulinemia needs to be tested.

Our data on the restraining ability of the RAGE inhibitors FPS-ZM1 and RAP in BC cells, in animal models, as well as in patient-derived samples provides solid pre-clinical validation on the feasibility of anti-RAGE strategies in breast malignancies during hyperinsulinemia. The observation that RAGE KO mice are viable and healthy, without any evident signs of altered embryonic development further points at RAGE as an actionable drug target [60]. In this regard, RAGE inhibition was shown to repress BC progression in vitro and in vivo in several independent investigations. For instance, in a transgenic mouse model of BC engineered to mimic the hyperactivation of RAGE signaling through the overexpression of the RAGE ligand S100A7, the administration of a RAGE neutralizing antibody hampered metastasis formation to the lung [61]. Adding to this, RAGE inhibition by FPS-ZM1 was able to restrain tumor formation and metastasis propagation, even in the absence of putative RAGE ligands [62]. In our in vitro models, we used concentrations of FPS-ZM1 (10 μM) that do not affect cell viability, thus allowing us to rule out any toxicity of the RAGE inhibitor. In fact, we observed only a slight and non-significant reduction in cell viability in our in vitro models stimulated with 2 μM and 10 μM FPS-ZM1. These concentrations of the drug were well tolerated, in accordance with previous studies indicating that concentrations of FPS-ZM1 ranging from 10 to 40 μM may be required to reach effective receptor blockade, depending on the experimental setting, without toxic effects [63–65].

Our results extend these findings and suggest that repressing RAGE signaling in Ins-rich milieu may represent a feasible and promising option to be warranted in further preclinical and clinical evaluation.

In non-cancer tissues like adipocytes, RAGE is involved in inflammation-dependent Ins resistance, a condition characterized by a reduced ability of Ins to activate IR/IRS1/AKT signaling [51]; in these contexts, the inhibition or ablation of RAGE restores Ins sensitivity and glucose homeostasis [51]. Therefore, in certain conditions targeting RAGE represents a mean to antagonize Ins resistance and re-prime Ins signaling in target tissues. Our data indicate that the pharmacological or genetic depletion of RAGE suppresses Ins signaling in BC cells, thus suggesting cell- and tissue-specific mechanisms involved in RAGE-dependent regulation of the IR pathway. It could be postulated that a fine-tuned balance between repression and stimulation of IR signaling may be mediated by RAGE in diverse physio-pathological conditions, perhaps in response to certain environmental stimuli such as oxidative and inflammatory stress. Evidence that ROS and inflammatory cytokines may behave differently in modulating Ins action in diverse physio-pathological contexts as part of a more complex spatio-temporal regulation has been provided extensively. This is the case for interleukin-1 (IL-1)β, whose time-restricted release is required for the post-prandial secretion of Ins and its action as hypoglycemic hormone [66]; on the other hand, a prolonged exposure to IL-1β prompts non-canonical pathways implicated in loss of Ins-mediated signals and ultimately Ins resistance [67]. Similarly, the generation of oxidative stress is required for IR activation by Ins, whereas chronic ROS generation plays a mounting role in the onset and maintaining of Ins resistance [26, 68].

Interestingly, in BC cells exposed to Ins, FPS-ZM1 repressed glucose utilization by the glycolytic pathway, as evidenced by the analysis of metabolic flux. These data were backed up by proteomic studies followed by pathway enrichment analyses, which showed that RAGE inhibition impairs the predicted up-regulation of the glycolytic pathway induced by Ins. On the other hand, the systemic glucose-lowering ability of Ins was not restrained by FPS-ZM1 in our allograft animal model, thus suggesting that the obliteration of RAGE signaling in hyperinsulinemic BC patients could be pursued without obtaining diabetic-like responses. Although we collected blood for the determination of glucose levels in our animal model, we did not measure circulating concentrations of Ins, therefore further investigations, possibly in more advanced pre-clinical models, are warranted to corroborate our findings. In this context, results from ongoing clinical trials, aimed at understanding whether repressing RAGE axis is beneficial at relieving both Ins resistance and BC progression, may contribute to build up critical knowledge that will turn useful in translational purposes [ClinicalTrials.gov identifier: NCT03092635].

Interestingly, we found that the inhibition of RAGE hampers not only Ins/IR signaling, but also its cross-talk with IGF-1R. Likewise, Ins was still able to convey stimulatory signals in IR KO cells, an effect likely mediated by IGF-1R and mitigated by FPS-ZM1. On the other hand, the ability of Ins to activate the oncogenic cascade IR/IRS1/AKT/CD1, and the inhibition of this pathway upon treatment with FPS-ZM1 did not require a functional IGF-1R, as evidenced using IGF-1R KO models. Further supporting the ability of RAGE inhibition to hamper the IR and IGF-1R-mediated effects, we demonstrated that FPS-ZM1 serves as a negative modulator of oncogenic signals prompted in response to both IGF-1 and IGF-2 in BC cells. Additional studies are required to address whether RAGE inhibition halts the signals mediated by both IR isoforms and if there’s evidence of a higher propensity to block IR-A versus IR-B-mediated signals and biological functions.

Furthermore, a more in depth evaluation of the potential of RAGE inhibition should be addressed in future investigations, in light of the exciting results from our proteomic studies and pathway enrichment analysis, showing that FPS-ZM1 suppresses the ability of Ins not only to stimulate the Ins pathway, but also several tumorigenic transduction cascades classically activated in response to Ins, such as estrogen signaling [69], PI3K/AKT signaling [14], RAS signaling [70], JAK/STAT signaling [14], Focal adhesion signaling [71] and ERBb signaling [73]. An additional important focus of future studies is the observation that RAGE inhibition mitigates the activation of stressful and inflammatory pathways previously associated with Ins-mediated action, including HIF-1 signaling [73] and NF-κB signaling [14].

Of note, the stimulatory cross-talk between IR and RAGE was evidenced also in CAFs obtained from BC patients, suggesting that blocking RAGE represents an appealing strategy to halt oncogenic signals initiated by Ins also in the breast tumor microenvironment. These findings extend previous studies on the ability of IIGFs to drive stimulatory responses mediated by CAFs [43]. Furthermore, our data extend the current knowledge on the ability of the RAGE axis to mediate relevant biological responses in CAFs, including their activation, the reprogramming of tumor metabolism, the regulation of mechano-transduction signaling, and the facilitation of metastatic switch [74–77]. As CAFs represent a relevant component of the tumor microenvironment implicated in complex biological responses such as angiogenesis, cell migration, invasion, inflammation, and immune evasion, our data provide novel evidence on the opportunity to control aberrant microenvironmental responses conducive to BC progression by means of RAGE targeting.

Collectively, these findings highlight a novel cross-talk between IR and RAGE in BC microenvironments characterized by high Ins levels. Our data indicate that targeting RAGE may be regarded as a novel opportunity to blunt disease progression in Ins-rich breast tumor microenvironments. Overall, our study provides an initial platform for further exploring the feasibility of RAGE-blocking strategies in BC patients, particularly when affected by obesity, pre-diabetes and/or diabetes.

### Supplementary Information

**Additional file 1**. **Fig. S1.** IR and RAGE correlation in ER-positive and ER-negative BC patients of the METABRIC cohort.

**Additional file 2**. **Fig. S2.** Stimulatory pathways activated by Ins in BC cells.

**Additional file 3**. **Fig. S3.** Mechanisms of RAGE and IR cooperation toward the activation of Ins_IR signaling.

**Additional file 4**. **Fig. S4.** Heatmaps of DEPs

**Additional file 5**.** Fig. S5.** Topological representation of MITHrIL outputs from proteomic analysis for the PI3K_AKT pathway

**Additional file 6**.** Fig. S6.** Characterization of CAFs and tumor homogenates

**Additional file 7**.** Fig. S7.** Full gels and blots images

**Additional file 8**.** Table S1**. Schematic representation of pathways affected by RAGE inhibition in Ins-stimulated MCF-7 cells, as evidenced by pathway enrichment analysis of proteomic outputs

**Additional file 9**. **Table S2**. Relevant clinical information and characteristics of patient-derived tumors used in the study

**Additional file 10**. Supplementary Materials and Methods.

## Data Availability

Data are available upon request addressed to the corresponding author.
