# Publisher Correction: RAGE inhibition blunts insulin-induced oncogenic signals in breast cancer

**DOI:** 10.1186/s13058-023-01689-2

**Published:** 2023-08-10

**Authors:** M. G. Muoio, M. Pellegrino, V. Rapicavoli, M. Talia, G. Scavo, V. Sergi, V. Vella, S. Pettinato, M. G. Galasso, R. Lappano, D. Scordamaglia, F. Cirillo, A. Pulvirenti, D. C. Rigiracciolo, M. Maggiolini, A. Belfiore, E. M. De Francesco

**Affiliations:** 1 Endocrinology, Department of Clinical and Experimental Medicine, Garibaldi-Nesima Hospital, University of Catania, 95122 Catania, Italy; 2 Department of Pharmacy, Health and Nutritional Sciences, University of Calabria, 87036 Rende, Italy; 3 Breast Unit Breast Surgery, Garibaldi-Nesima Hospital, 95122 Catania, Italy; 4 Pathological Anatomy Unit, Garibaldi-Nesima Hospital, 95122 Catania, Italy; 5 Bioinformatics Unit, Department of Clinical and Experimental Medicine, University of Catania, 95131 Catania, Italy; 6 Department of Experimental Oncology, IEO, European Institute of Oncology IRCCS, Via Adamello 16, 20139 Milan, Italy

**Correction: Breast Cancer Research (2023) 25:84** 10.1186/s13058-023-01686-5

The original publication of this article contained Table 1 which did not belong to the publication.

The table has been removed from the original publication, the publisher apologizes for the inconvenience caused to the authors and readers.

