# Supplementary material for: RAGE inhibition blunts insulin-induced oncogenic signals in breast cancer
Source: Breast Cancer Res. 2023 Jul 17;25:84. doi: 10.1186/s13058-023-01686-5 (PMC10351154; doi:10.1186/s13058-023-01686-5)
Supplement: Supplementary file 1 — Additional file 1. Fig. S1. IR and RAGE correlation in ER-positive and ER-negative BC patients of the METABRIC cohort. [file 13058_2023_1686_MOESM1_ESM.docx]

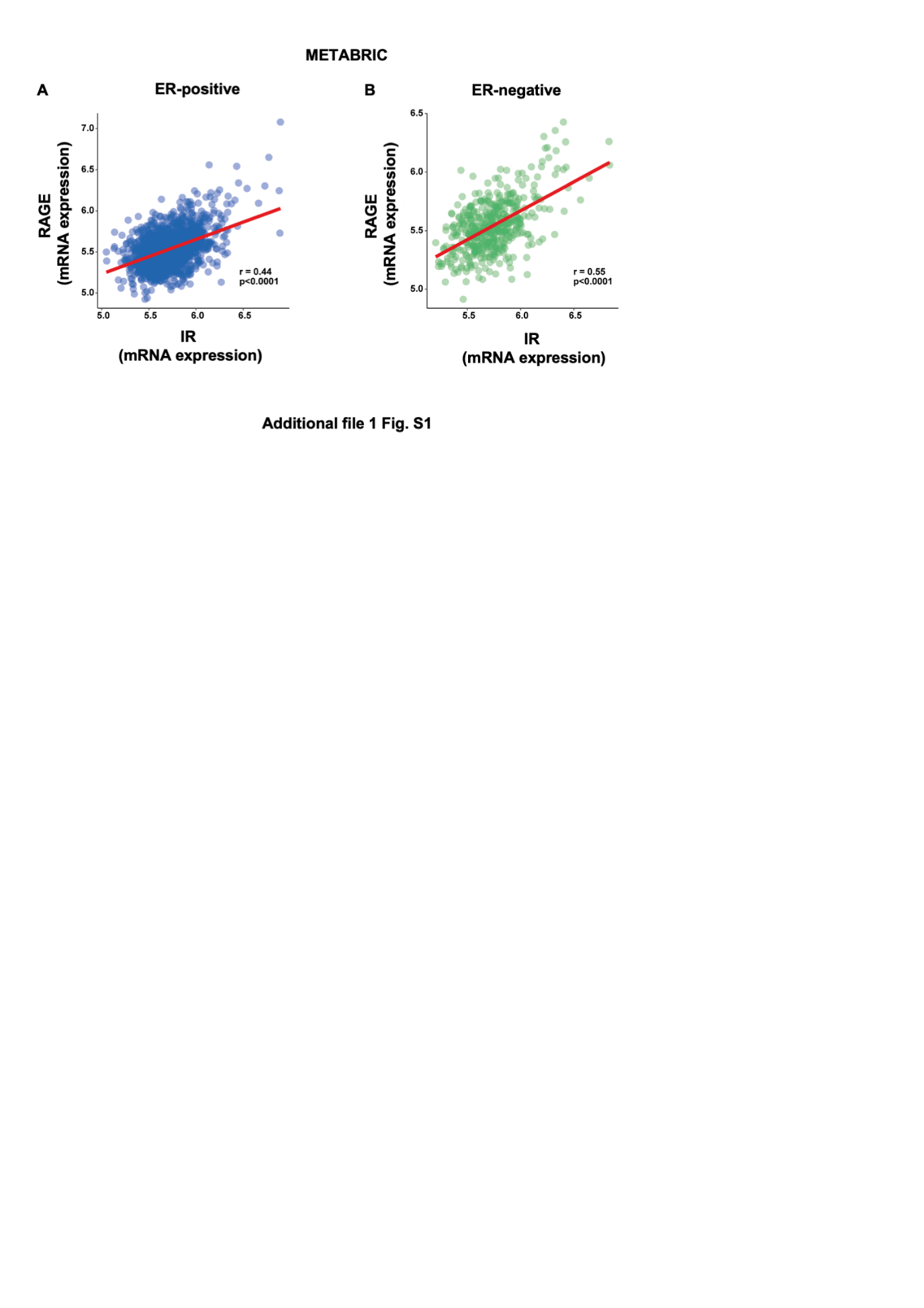


**Additional file 1 Fig.S1 IR and RAGE correlation in ER-positive and ER-negative BC patients of the METABRIC cohort.** Scatter plots showing the correlation between RAGE and IR expression levels in ER-positive (n. 1459) (A) and ER-negative (n. 445) (B) BC patients of the METABRIC cohort. The Pearson correlation coefficients (r) and the relative p-values are shown in the panels.
