# Supplementary material for: RAGE inhibition blunts insulin-induced oncogenic signals in breast cancer
Source: Breast Cancer Res. 2023 Jul 17;25:84. doi: 10.1186/s13058-023-01686-5 (PMC10351154; doi:10.1186/s13058-023-01686-5)
Supplement: Supplementary file 2 — Additional file 2. Fig. S2. Stimulatory pathways activated by Ins in BC cells. [file 13058_2023_1686_MOESM2_ESM.docx]

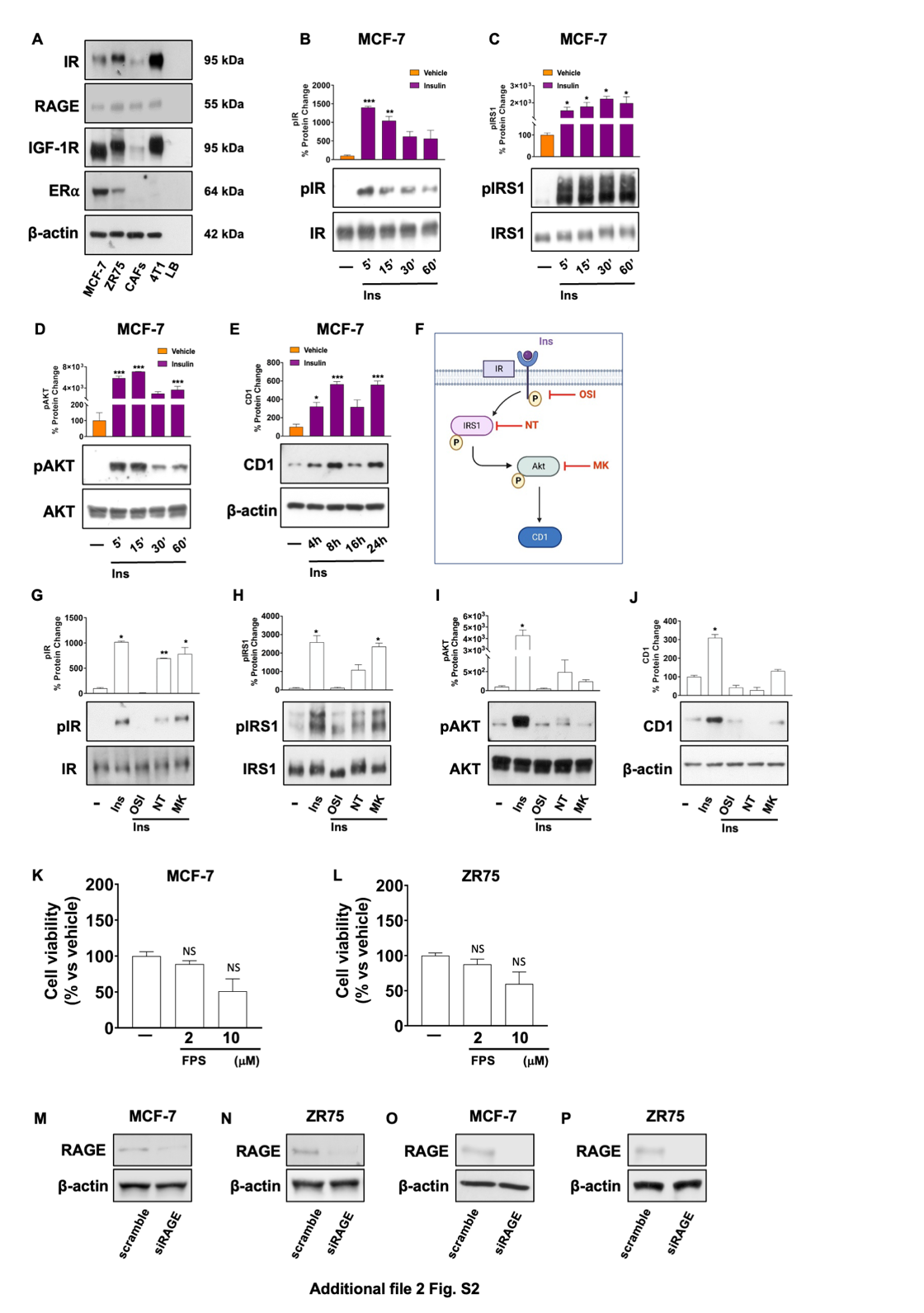


**Fig. S2 Stimulatory pathways activated by Ins in BC cells.** Evaluation of IR, RAGE, IGF-1R and ERα protein expression by western blotting in MCF-7, ZR75, CAFs and 4T1 cells. Lysis buffer (LB) without proteins was used in lane 5 as negative control (A). Representative immunoblots showing the activation of IR (Y1135/1136) (B), IRS1 (Y612) (C), AKT (S473) (D) and the upregulation of CD1 (E) protein expression in MCF-7 cells treated with Ins (20 nM, as indicated). Schematic representation of the IR/IRS1/AKT/CD1 pathway activated by Ins in MCF-7 cells. Pharmacological inhibitors of putative mediators are shown in red (F). The phosphorylation of IR (Y1135/1136) (G), IRS1 (Y612) (H), and AKT (S473) (I) induced by Ins (20 nM, 15 min) is prevented in the presence of the IR inhibitor OSI-906 (OSI, 10 μM) (G), the IRS-1/2 selective inhibitor NT157 (NT 300 nM) (H), as well as the AKT inhibitor MK-2206 (MK 500 nM) (I). The up-regulation of CD1 protein expression was prevented in MCF-7 cells treated with Ins (20 nM, 4 h), alone and in combination with OSI (10 µM), NT (300 nM), and MK (500 nM) (J). Evaluation of cell viability by SRB assay in MCF-7 (K) and ZR75 (L) cells stimulated with 2 mM and 10 mM FPS-ZM1 for 72 h. Efficiency of RAGE silencing in MCF-7 (~~K, M~~ M, O) and ZR75 ~~(L, N~~ N, P) cells, relative to data shown in Figure 3 A-H. Total proteins and β-actin serve as loading control, as indicated. Data shown are mean ± SEM of at least three independent experiments. (*) p < 0.05; (**) p < 0.01; (***) p < 0.001.
