# Supplementary material for: RAGE inhibition blunts insulin-induced oncogenic signals in breast cancer
Source: Breast Cancer Res. 2023 Jul 17;25:84. doi: 10.1186/s13058-023-01686-5 (PMC10351154; doi:10.1186/s13058-023-01686-5)
Supplement: Supplementary file 3 — Additional file 3. Fig. S3. Mechanisms of RAGE and IR cooperation toward the activation of Ins_IR signaling. [file 13058_2023_1686_MOESM3_ESM.docx]

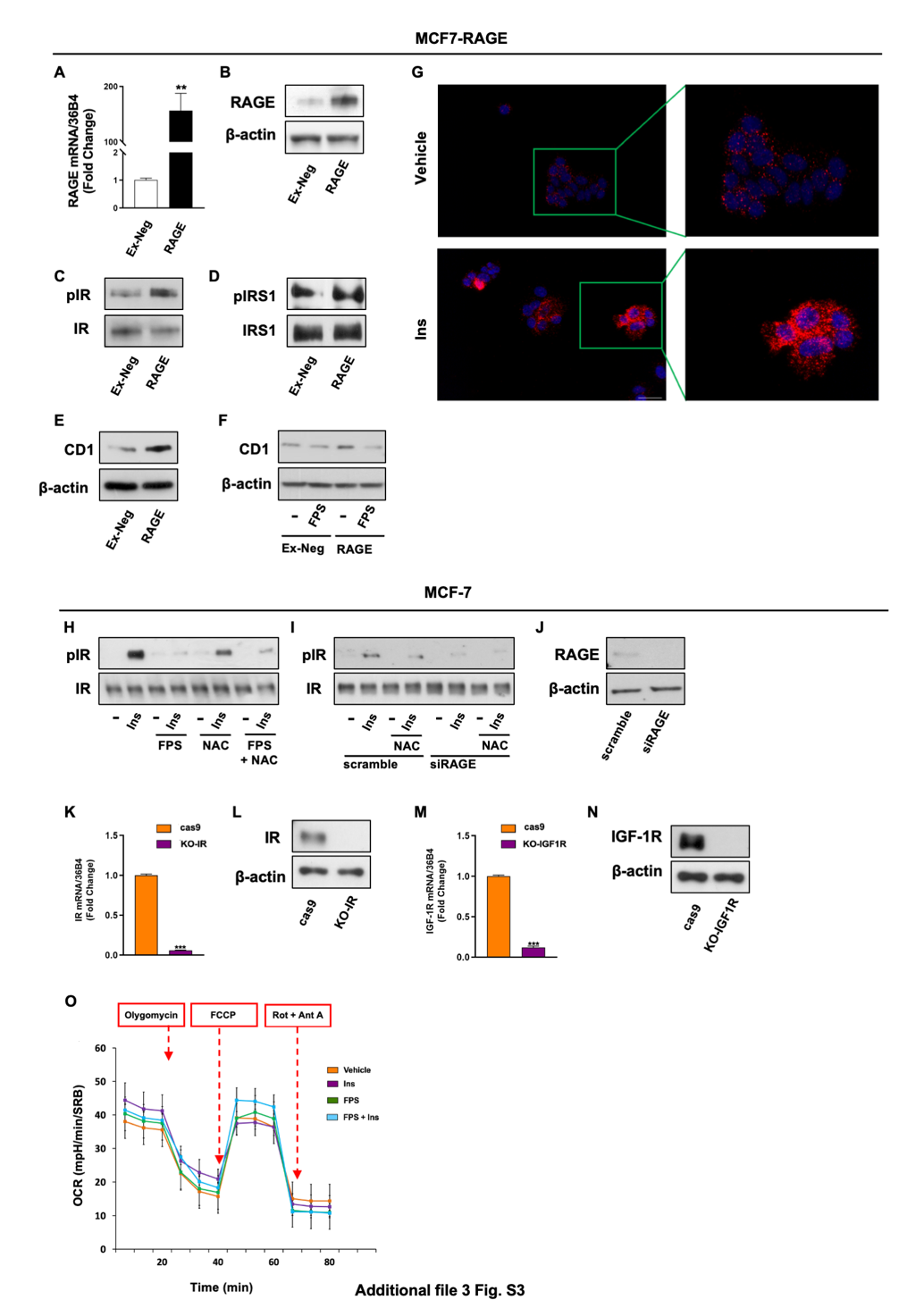


**Fig. S3 Mechanisms of RAGE and IR cooperation toward the activation of Ins/IR signaling.** Efficacy of RAGE overexpression obtained by lentiviral transduction in MCF7-Ex Neg (control) and MCF7- RAGE (overexpressing RAGE) cells, as evaluated by qRT-PCR (A) and western blotting (B). Evaluation of pIR (Y1135/1136) (C), pIRS1 (Y612) (D) and CD1 (E) protein expression in MCF7-Ex Neg (control) and MCF7- RAGE (overexpressing RAGE) cells subjected to serum-starvation for 24 h. FPS-ZM1 (10 μM, 24 h) prevents the increase of CD1 in MCF7-RAGE cells (F). In situ Proximity ligation assay in MCF7-RAGE cells treated with vehicle (-) or Ins (20 nM, 5 min). Red fluorescence indicates the membrane proximity of IR and RAGE (< 30 – 40 nm). Nuclei are stained by DAPI (blue fluorescence) (G). Evaluation of pIR (Y1135/1136) in MCF-7 cells treated with Ins (20 nM, 15 min), alone and in combination with FPS-ZM1 (10 μM, 24 h) and the ROS scavenger NAC (3 mM, 24 h) (~~G~~ H). Evaluation of pIR (Y1135/1136) in MCF-7 cells transfected with siRAGE or non-targeting scramble control (24 h) and then treated with Ins (20 nM, 15 min), alone and in combination with NAC (3 mM, 24 h) (~~H~~ I). Efficacy of RAGE silencing (~~I~~ J). Efficacy of IR (K-L) and IGF-1R (M-N) knock-out, as evaluated by qRT-PCR and western blotting in MCF-7 cells. Representative tracing of Oxygen Consumption Rate (OCR) by Seahorse analysis in MCF-7 cells treated with Ins (20 nM, 8 h) alone and in combination with FPS-ZM1 (10 μM, 24 h) (O). In western blotting experiments, total proteins and β-actin serve as loading control. In qRT-PCR experiments, values are normalized to the 36B4 gene expression and shown as fold changes of mRNA expression in cells expressing the empty vector (MCF7-Ex-Neg) compared to cells overexpressing RAGE (MCF7-RAGE). Data shown are mean ± SEM of at least three independent experiments performed in duplicate. (**) p < 0.01; (***) p < 0.001.
