# Supplementary material for: RAGE inhibition blunts insulin-induced oncogenic signals in breast cancer
Source: Breast Cancer Res. 2023 Jul 17;25:84. doi: 10.1186/s13058-023-01686-5 (PMC10351154; doi:10.1186/s13058-023-01686-5)
Supplement: Supplementary file 4 — Additional file 4. Fig. S4. Heatmaps of DEPs [file 13058_2023_1686_MOESM4_ESM.docx]

**
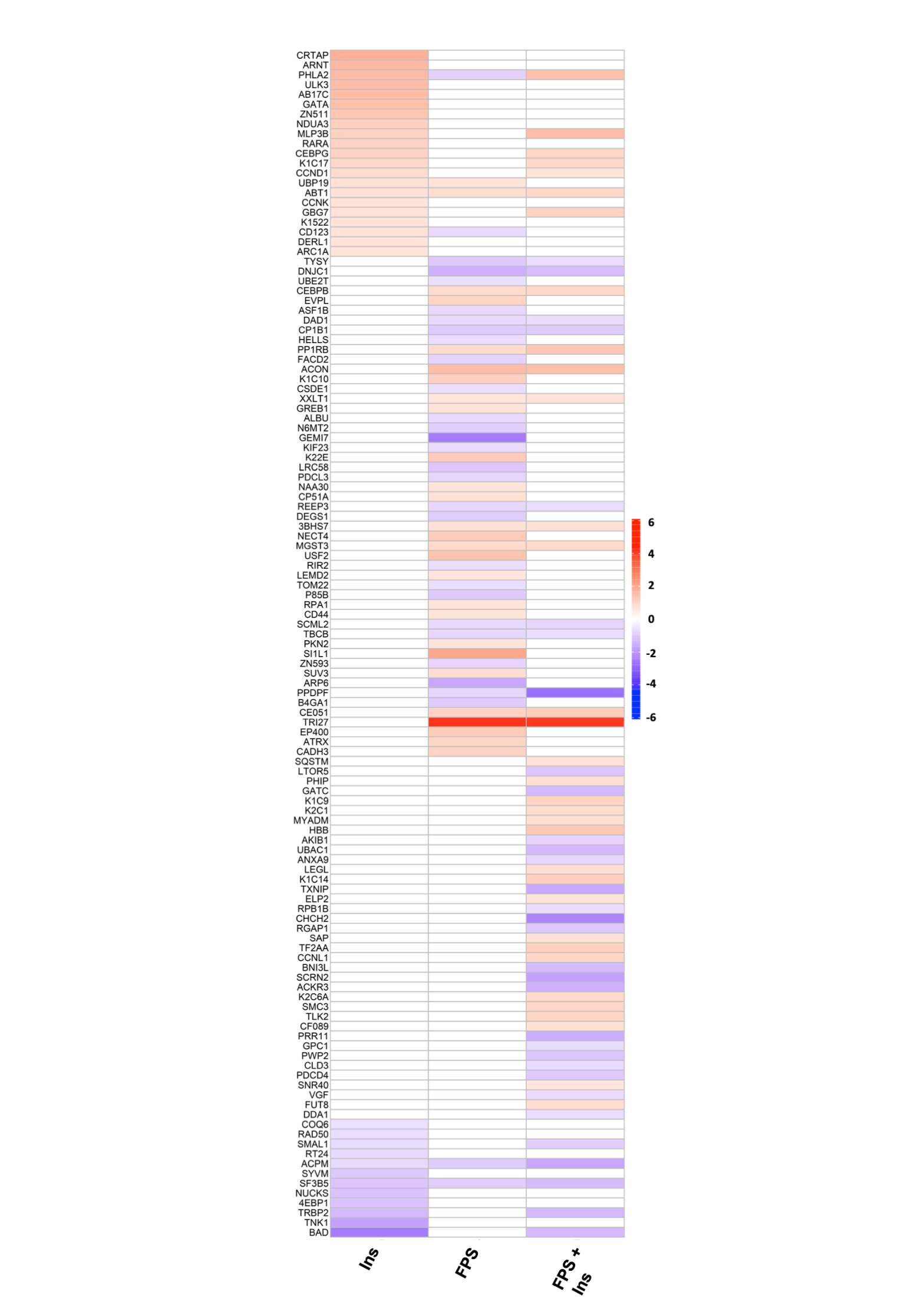
**

**Fig. S4 Heatmaps of DEPs.** Heatmap of differentially expressed proteins (DEPs) (p < 0.05) ordered by Ins in all experimental groups compared to vehicle-treated cells. Red up-regulation, blue down-regulation.
