# Supplementary material for: RAGE inhibition blunts insulin-induced oncogenic signals in breast cancer
Source: Breast Cancer Res. 2023 Jul 17;25:84. doi: 10.1186/s13058-023-01686-5 (PMC10351154; doi:10.1186/s13058-023-01686-5)
Supplement: Supplementary file 5 — Additional file 5. Fig. S5. Topological representation of MITHrIL outputs from proteomic analysis for the PI3K_AKT pathway [file 13058_2023_1686_MOESM5_ESM.docx]

**
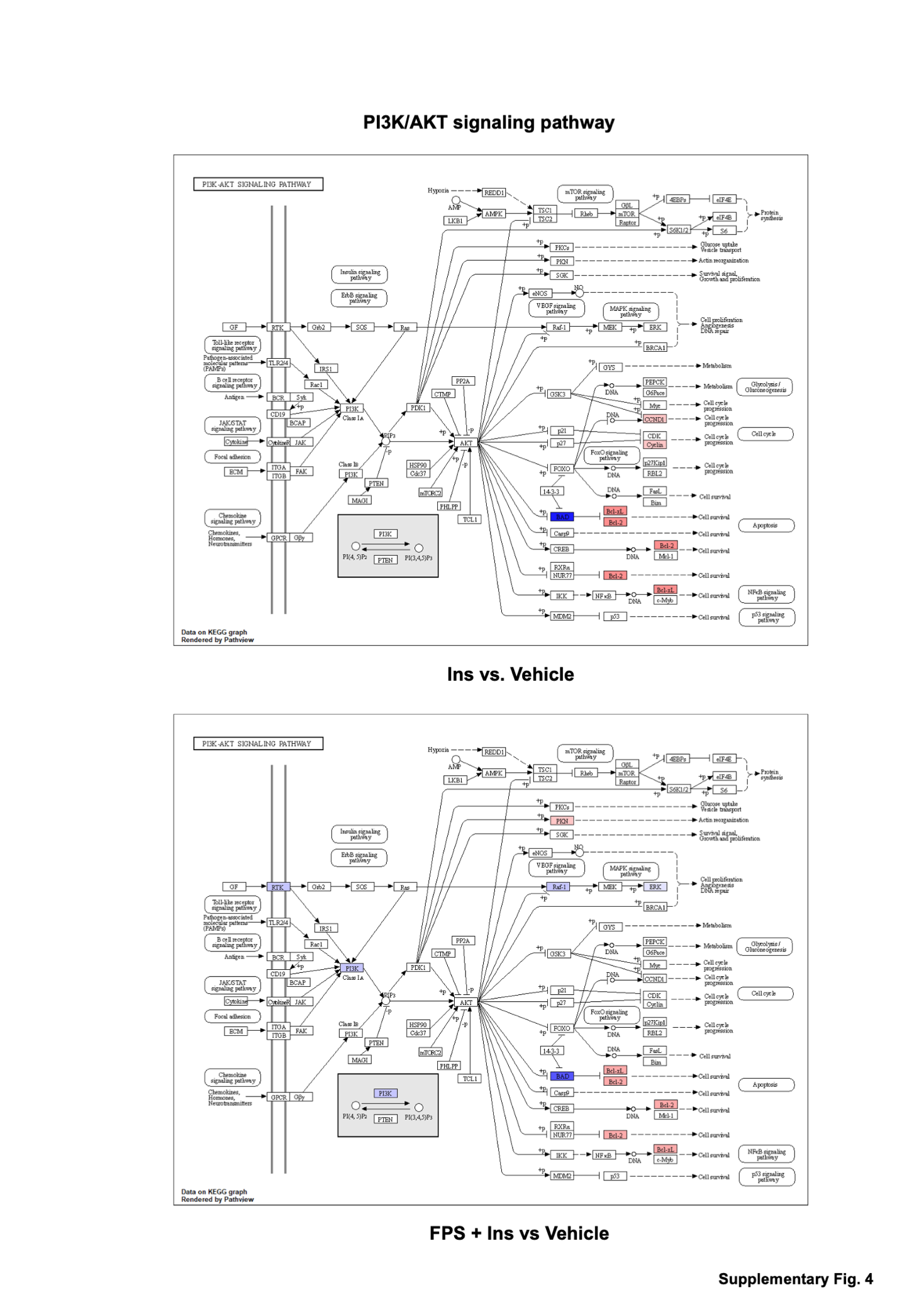
**

**Fig. S5 Topological representation of MITHrIL outputs from proteomic analysis for the PI3K/AKT pathway**. Nodes associated with cell proliferation, survival and antiapoptotic effects are upregulated by Ins, whereas FPS-ZM1 attenuates this effect. Red indicates up-regulation; blue indicates down-regulation.
