# Supplementary material for: RAGE inhibition blunts insulin-induced oncogenic signals in breast cancer
Source: Breast Cancer Res. 2023 Jul 17;25:84. doi: 10.1186/s13058-023-01686-5 (PMC10351154; doi:10.1186/s13058-023-01686-5)
Supplement: Supplementary file 6 — Additional file 6. Fig. S6. Characterization of CAFs and tumor homogenates [file 13058_2023_1686_MOESM6_ESM.docx]

**
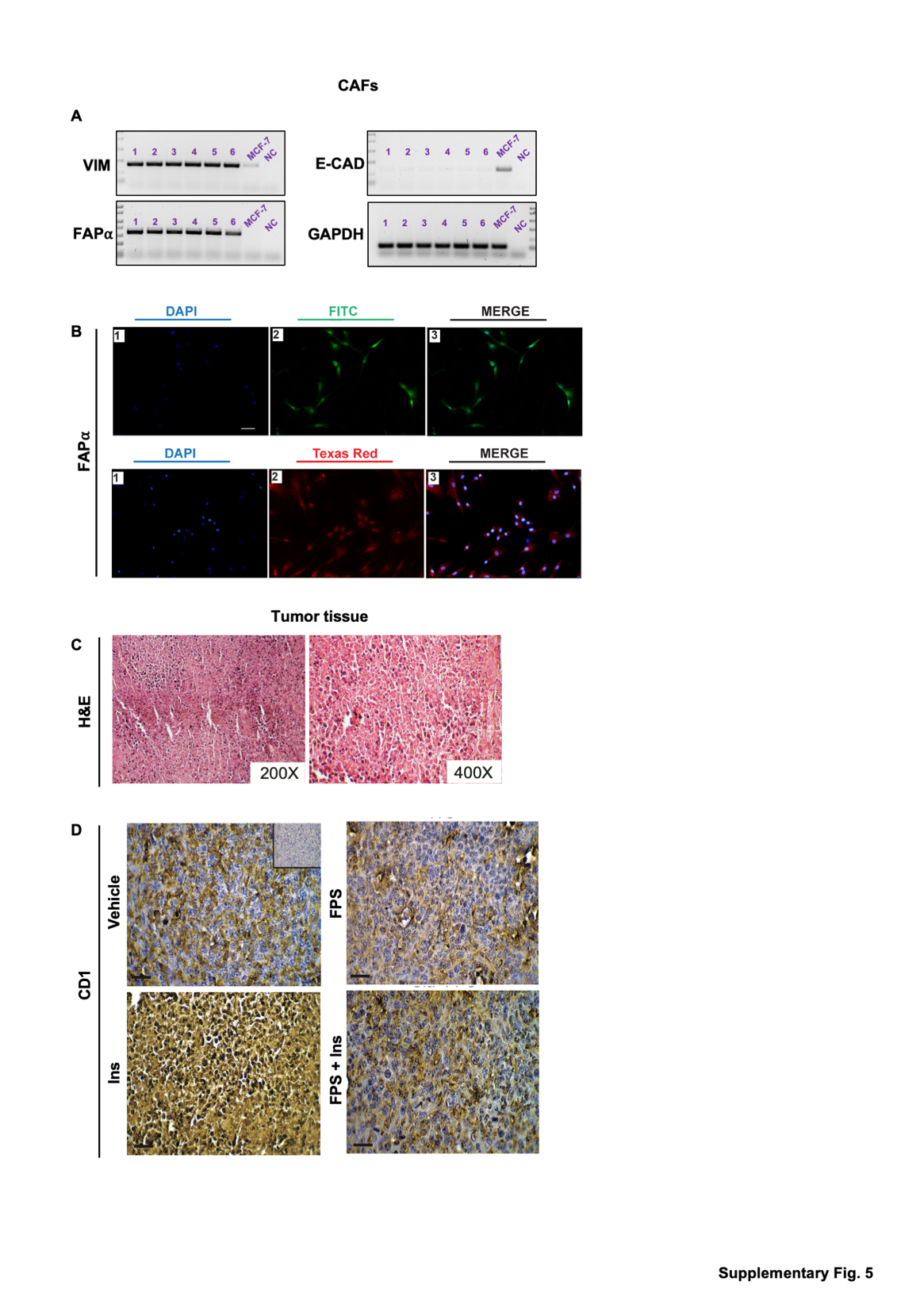
**

**Fig. S6 Characterization of CAFs and tumor homogenates.** Evaluation of Vimentin (VIM), E-cadherin (E-CAD) and FAPα mRNA expression by RT-PCR in six populations of CAFs derived from BC patients and in MCF-7 cells. Relevant clinical information and characteristics of patient-derived tumors used for CAFs isolation are reported in Supplementary Table 2. GAPDH serves as housekeeping gene. NC is negative control (A). Evaluation of FAPα protein expression by immunofluorescent microscopy in CAFs. FAPα accumulation is evidenced using two secondary antibodies emitting in the FITC channel or in the Texas Red channel, as indicated in Materials and Methods. Nuclei are stained by DAPI (blue signal) (B). Representative images from tumors that at the end of the treatments were formalin fixed, paraffin embedded, sectioned and stained with hematoxylin and eosin Y (H&E). 200x and 400x magnifications are shown (C). Evaluation of CD1 protein expression by IHC analysis in tumors from mice treated with Ins, alone and in combination with FPS-ZM1 for 28 days (D). After tumor removal, tissues were formalin fixed, paraffin embedded, sectioned and incubated with a mouse monoclonal antibody directed against CD1 that appears as a brown staining. Nuclei are counterstained with hematoxylin. Scale bar: 25 *µ*m.
