# Supplementary material for: RAGE inhibition blunts insulin-induced oncogenic signals in breast cancer
Source: Breast Cancer Res. 2023 Jul 17;25:84. doi: 10.1186/s13058-023-01686-5 (PMC10351154; doi:10.1186/s13058-023-01686-5)

Additional file 7 Fig. S7 Full gels and blots images

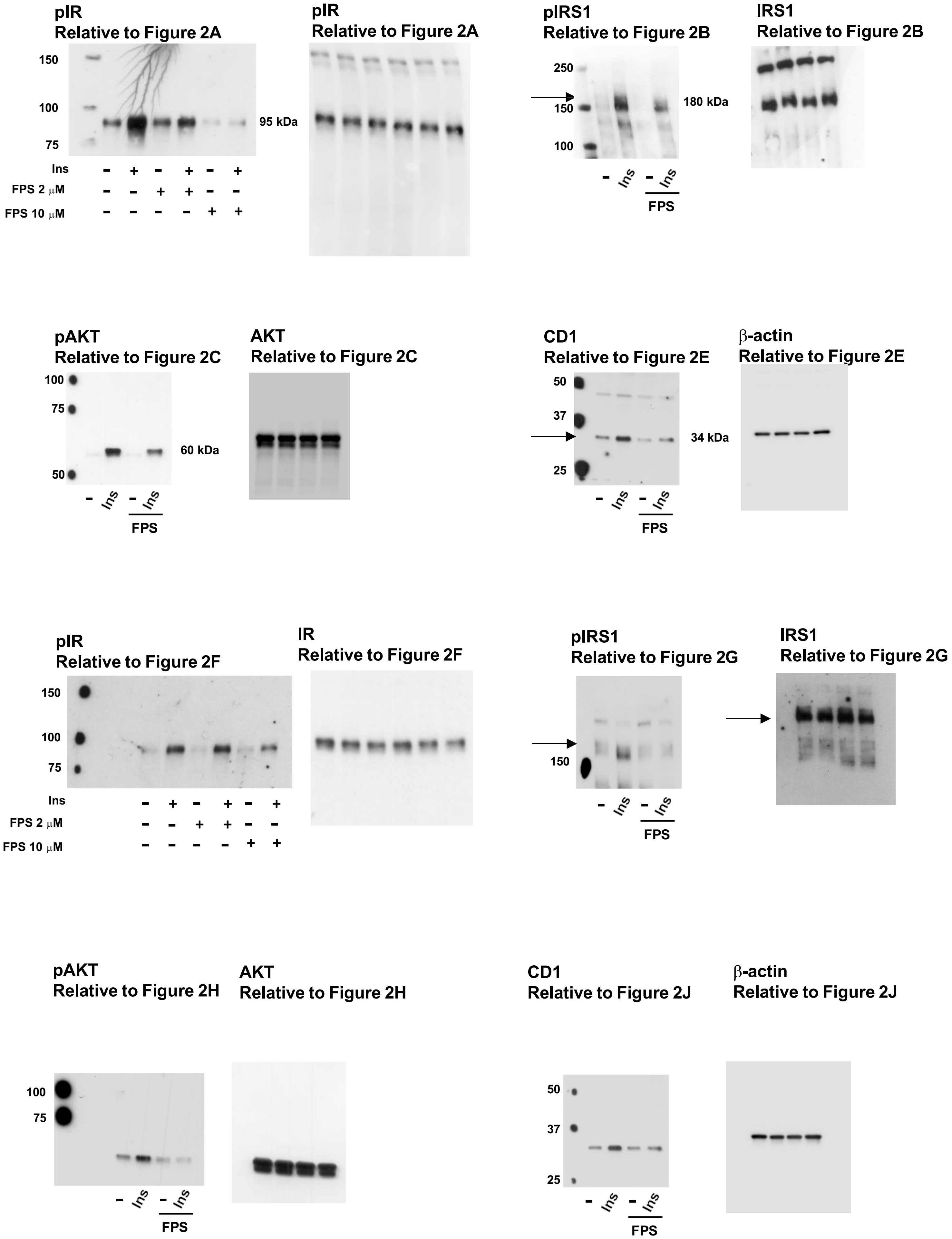

pIR
Relative to Figure 2K

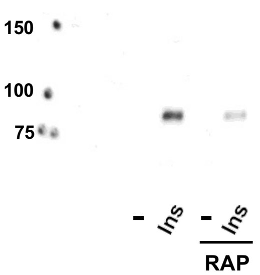

IR
Relative to Figure 2K

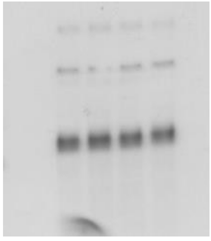

pIRS1
Relative to Figure 2L

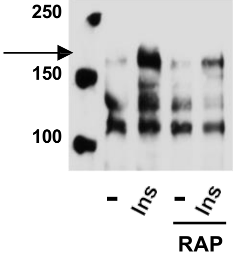

IRS1
Relative to Figure 2L

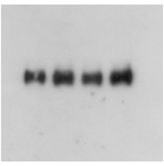

pAKT
Relative to Figure 2M

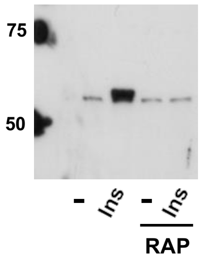

AKT
Relative to Figure 2M

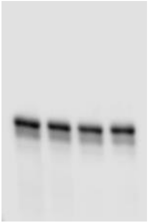

CD1
Relative to Figure 2N

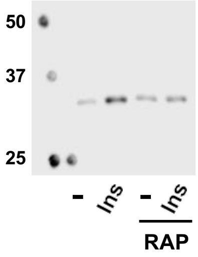

β-actin
Relative to Figure 2N

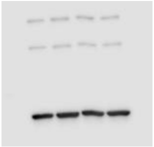

pIR
Relative to Figure 2O

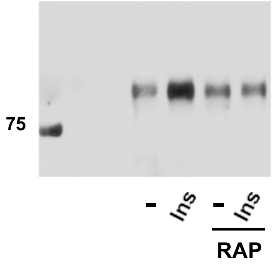

IR
Relative to Figure 2O

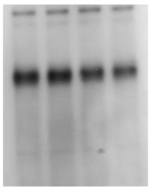

pIRS1
Relative to Figure 2P

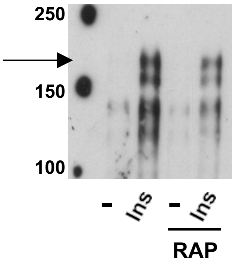

IRS1
Relative to Figure 2P

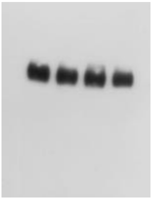

pAKT
Relative to Figure 2Q

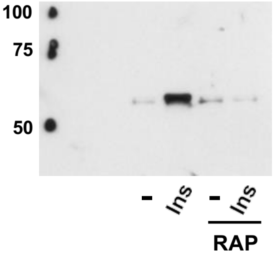

AKT
Relative to Figure 2Q

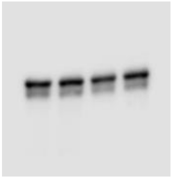

CD1
Relative to Figure 2R

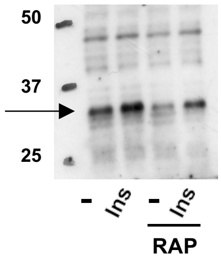

β-actin
Relative to Figure 2R

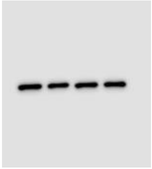

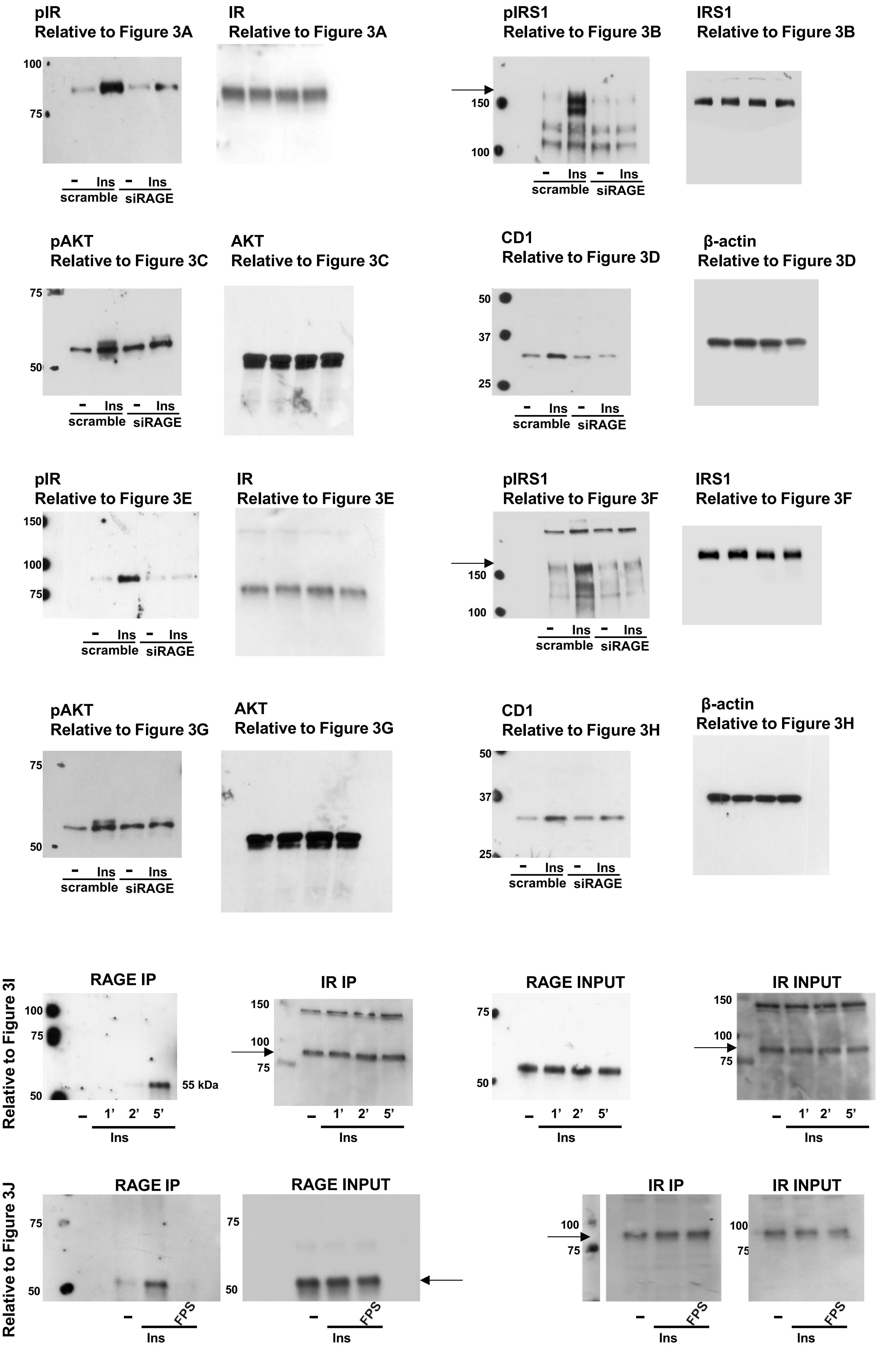
pIR
Relative to Figure 3A
100
75
- Ins - Ins
scramble siRAGE
IR
Relative to Figure 3A
pIRS1
Relative to Figure 3B
150
100
- Ins - Ins
scramble siRAGE
IRS1
Relative to Figure 3B
pAKT
Relative to Figure 3C
75
50
- Ins - Ins
scramble siRAGE
AKT
Relative to Figure 3C
CD1
Relative to Figure 3D
50
37
25
- Ins - Ins
scramble siRAGE
β-actin
Relative to Figure 3D
pIR
Relative to Figure 3E
150
100
75
- Ins - Ins
scramble siRAGE
IR
Relative to Figure 3E
pIRS1
Relative to Figure 3F
150
100
- Ins - Ins
scramble siRAGE
IRS1
Relative to Figure 3F
pAKT
Relative to Figure 3G
75
50
- Ins - Ins
scramble siRAGE
AKT
Relative to Figure 3G
CD1
Relative to Figure 3H
50
37
25
- Ins - Ins
scramble siRAGE
β-actin
Relative to Figure 3H
Relative to Figure 3I
RAGE IP
100
75
50
55 kDa
- 1' 2' 5'
Ins
IR IP
150
100
75
- 1' 2' 5'
Ins
RAGE INPUT
75
50
- 1' 2' 5'
Ins
IR INPUT
150
100
75
- 1' 2' 5'
Ins
Relative to Figure 3J
RAGE IP
75
50
- FPS
Ins
RAGE INPUT
75
50
- FPS
Ins
IR IP
100
75
- FPS
Ins
IR INPUT
100
75
- FPS
Ins

pIGF-1R/pIR
Relative to Figure 4A

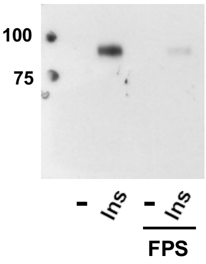

IGF1R
Relative to Figure 4A

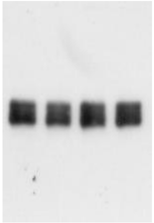

pIRS1
Relative to Figure 4B

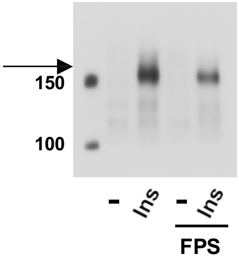

IRS1
Relative to Figure 4B

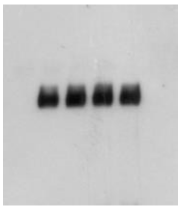

pAKT
Relative to Figure 4C

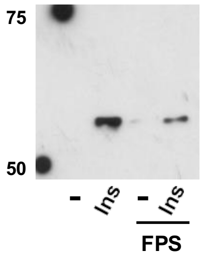

AKT
Relative to Figure 4C

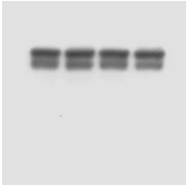

CD1
Relative to Figure 4D

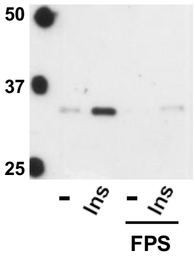

β-actin
Relative to Figure 4D

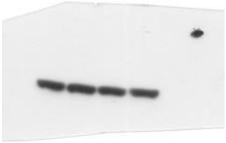

pIR/pIGF-1R
Relative to Figure 4E

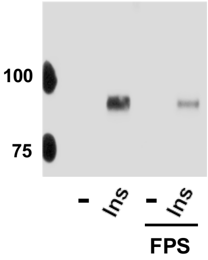

IR
Relative to Figure 4E

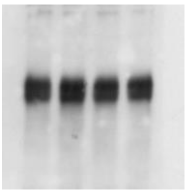

pIRS1
Relative to Figure 4F

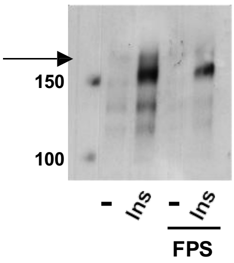

IRS1
Relative to Figure 4F

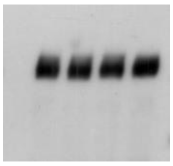

pAKT
Relative to Figure 4G

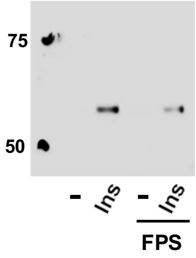

AKT
Relative to Figure 4G

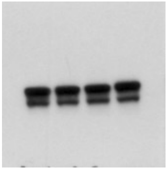

CD1
Relative to Figure 4H

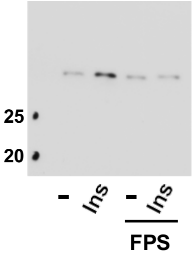

β-actin
Relative to Figure 4H

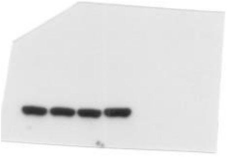

pIR/pIGF-1R
Relative to Figure 4I

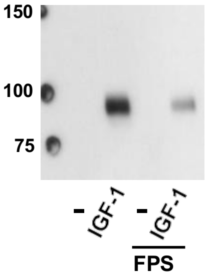

IGF-1R
Relative to Figure 4I

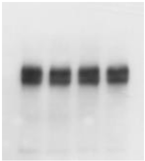

pIRS1
Relative to Figure 4J

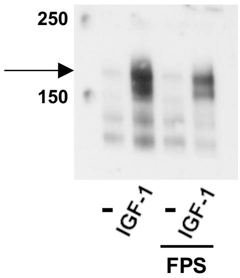

IRS1
Relative to Figure 4J

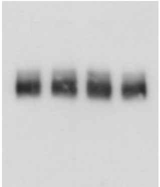

pAKT
Relative to Figure 4K

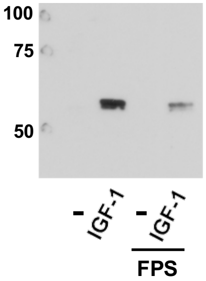

AKT
Relative to Figure 4K

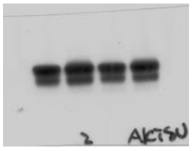

CD1
Relative to Figure 4L

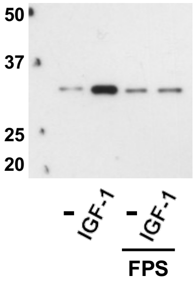

β-actin
Relative to Figure 4L

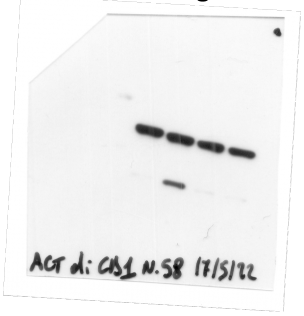

pIR/pIGF-1R
Relative to Figure 4M

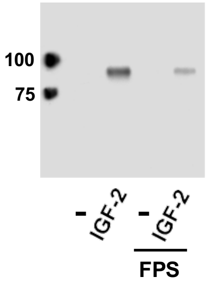

IGF-1R
Relative to Figure 4M

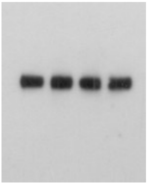

pIRS1
Relative to Figure 4N

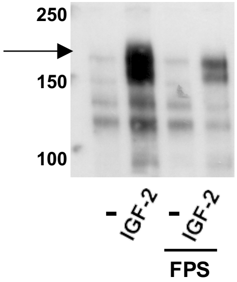

IRS1
Relative to Figure 4N

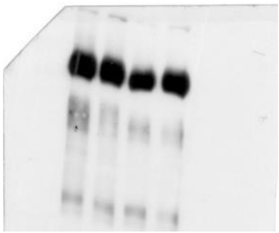

pAKT
Relative to Figure 4O

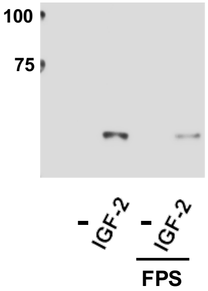

AKT
Relative to Figure 4O

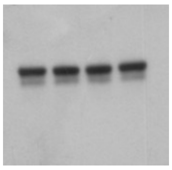

CD1
Relative to Figure 4P

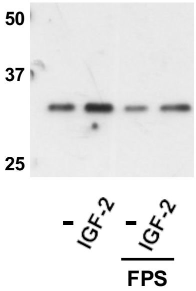

β-actin
Relative to Figure 4P

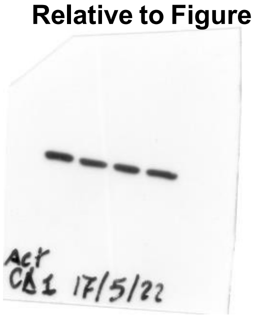

RAGE
Relative to Figure 7E

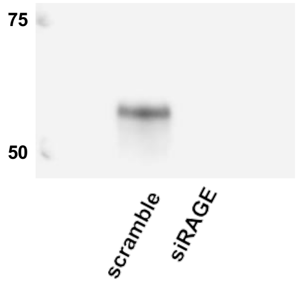

β-actin
Relative to Figure 7E

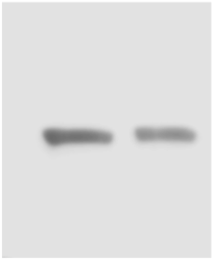

pIR
Relative to Figure 9A

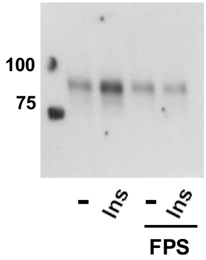

IR
Relative to Figure 9A

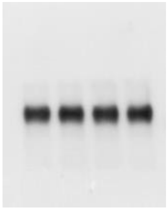

pIRS1
Relative to Figure 9B

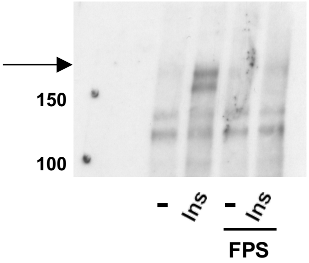

IRS1
Relative to Figure 9B

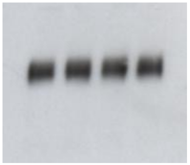

pAKT
Relative to Figure 9C

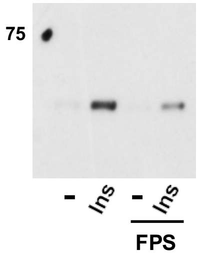

AKT
Relative to Figure 9C

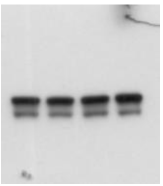

CYR61
Relative to Figure 9D

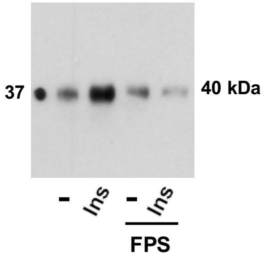

β-actin
Relative to Figure 9D

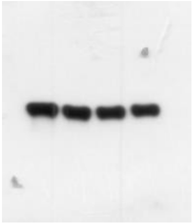

pIR
Relative to Figure 9F

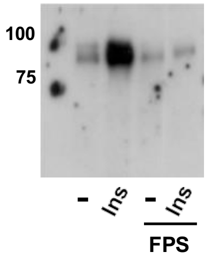

IR
Relative to Figure 9F

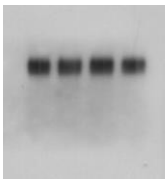

pIRS1
Relative to Figure 9G

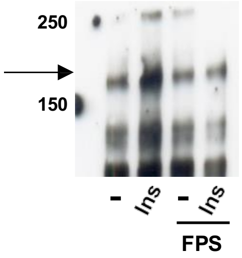

IRS1
Relative to Figure 9G

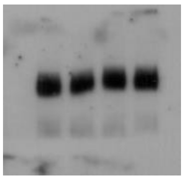

pAKT
Relative to Figure 9H

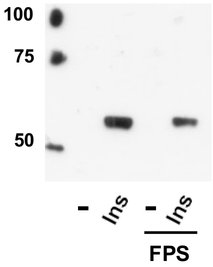

AKT
Relative to Figure 9H

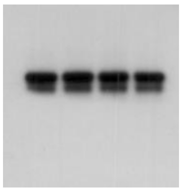

CD1
Relative to Figure 9I

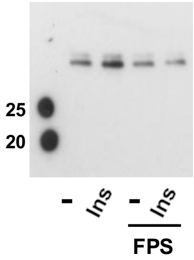

β-actin
Relative to Figure 9I

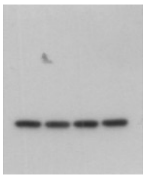

CD1
Relative to Figure 10E

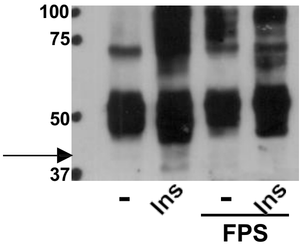

β-actin
Relative to Figure 10E

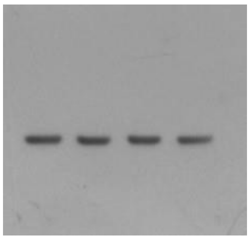

IR
Relative to Figure S2A

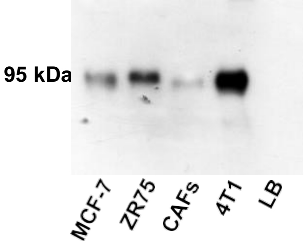

RAGE
Relative to Figure S2A

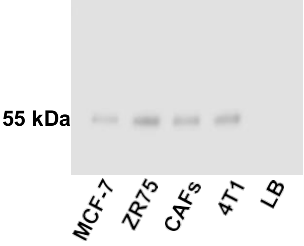

IGF-1R
Relative to Figure S2A

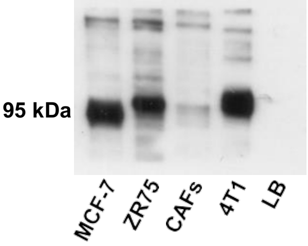

ER*a*
Relative to Figure S2A

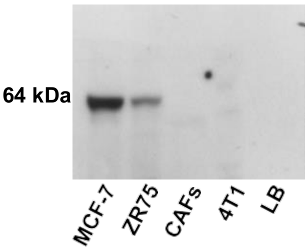

β-actin
Relative to Figure S2A

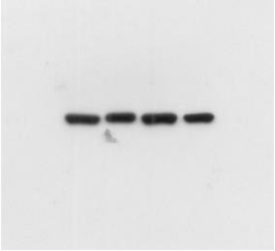

pIR
Relative to Figure S2B

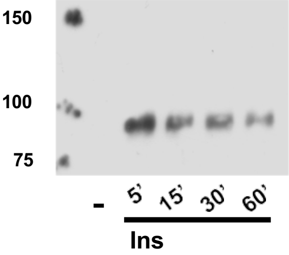

IR
Relative to Figure S2B

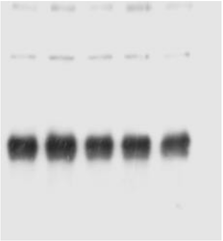

pIRS1
Relative to Figure S2C

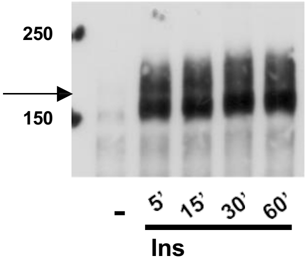

IRS1
Relative to Figure S2C

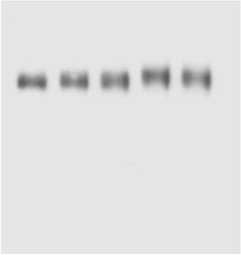

pAKT
Relative to Figure S2D

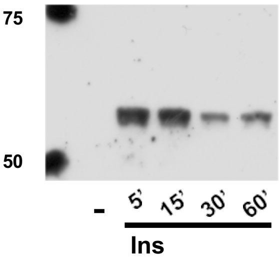

AKT
Relative to Figure S2D

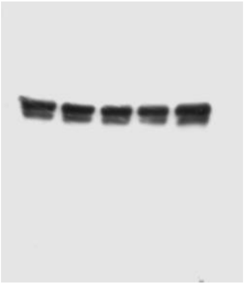

CD1
Relative to Figure S2E

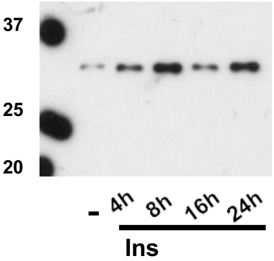

β-actin
Relative to Figure S2E

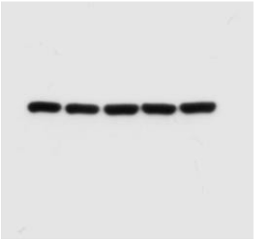

pIR
Relative to Figure S2G

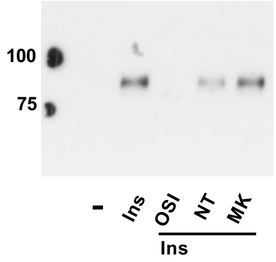

IR
Relative to Figure S2G

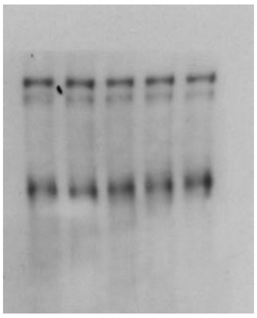

pIRS1
Relative to Figure S2H

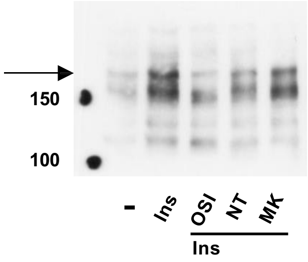

IRS1
Relative to Figure S2H

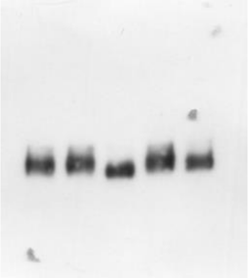

pAKT
Relative to Figure S2I

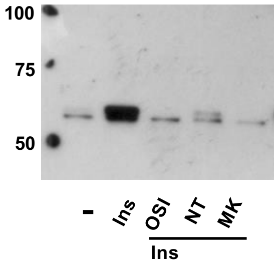

AKT
Relative to Figure S2I

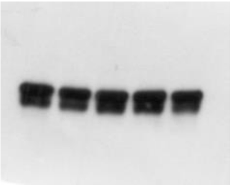

CD1
Relative to Figure S2J

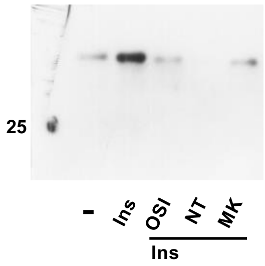

β-actin
Relative to Figure S2J

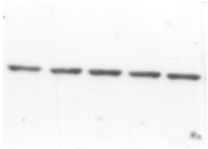

RAGE
Relative to Figure S2M

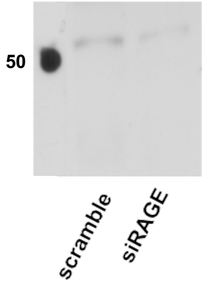

β-actin
Relative to Figure S2M

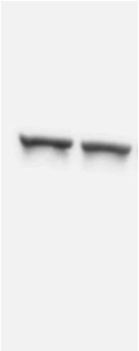

RAGE
Relative to Figure S2N

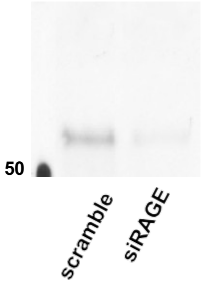

β-actin
Relative to Figure S2N

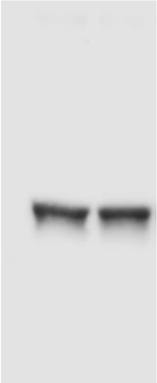

RAGE
Relative to Figure S2O

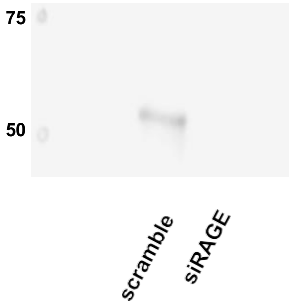

β-actin
Relative to Figure S2O

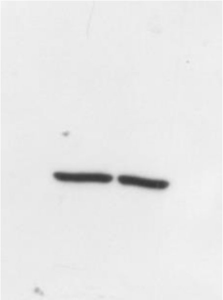

RAGE
Relative to Figure S2P

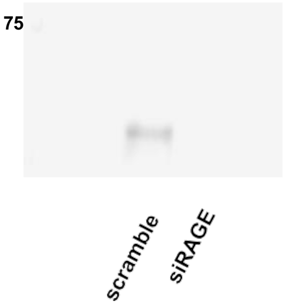

β-actin
Relative to Figure S2P

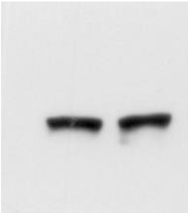

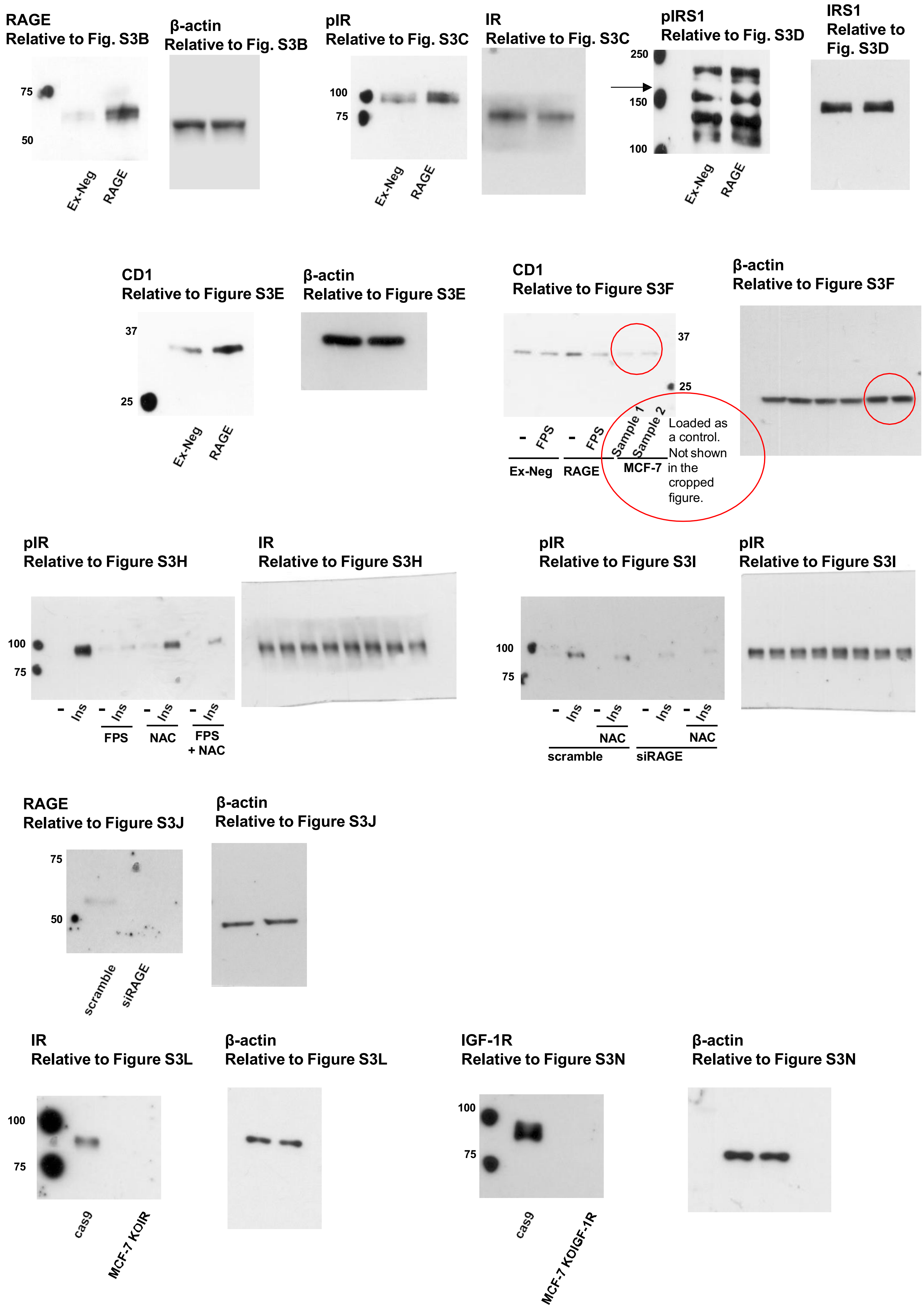

RAGE
Relative to Fig. S3B
75
50
Ex-Neg
RAGE
β-actin
Relative to Fig. S3B
pIR
Relative to Fig. S3C
100
75
Ex-Neg
RAGE
IR
Relative to Fig. S3C
pIRS1
Relative to Fig. S3D
250
150
100
Ex-Neg
RAGE
IRS1
Relative to
Fig. S3D
CD1
Relative to Figure S3E
37
25
Ex-Neg
RAGE
β-actin
Relative to Figure S3E
CD1
Relative to Figure S3F
37
25
- FPS - FPS Sample 1 Sample 2
Ex-Neg
RAGE
MCF-7
Loaded as a control. Not shown in the cropped figure.
β-actin
Relative to Figure S3F
pIR
Relative to Figure S3H
100
75
- Ins - Ins - Ins - Ins
FPS
NAC
FPS + NAC
IR
Relative to Figure S3H
pIR
Relative to Figure S3I
100
75
- Ins - Ins - Ins - Ins
NAC
NAC
scramble
siRAGE
pIR
Relative to Figure S3I
RAGE
Relative to Figure S3J
75
50
scramble
siRAGE
β-actin
Relative to Figure S3J
IR
Relative to Figure S3L
100
75
cas9
MCF-7 KOIR
β-actin
Relative to Figure S3L
IGF-1R
Relative to Figure S3N
100
75
cas9
MCF-7 KOIGF-1R
β-actin
Relative to Figure S3N

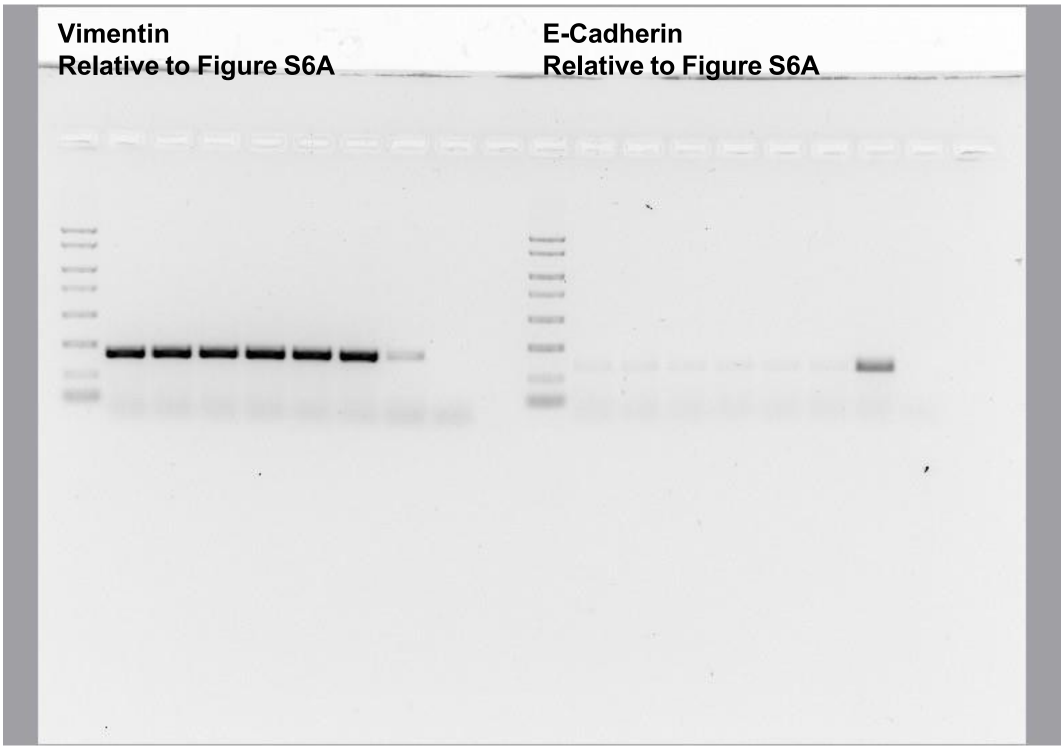

FAPα
Relative to Figure S6A

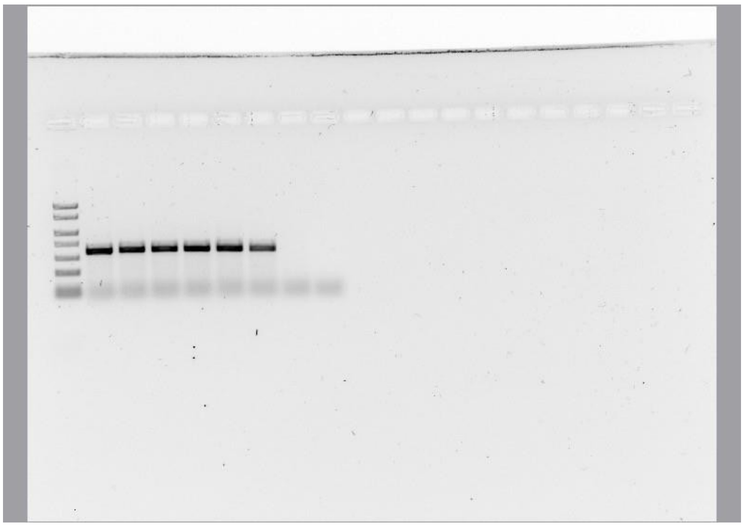

GAPDH
Relative to Figure S6A

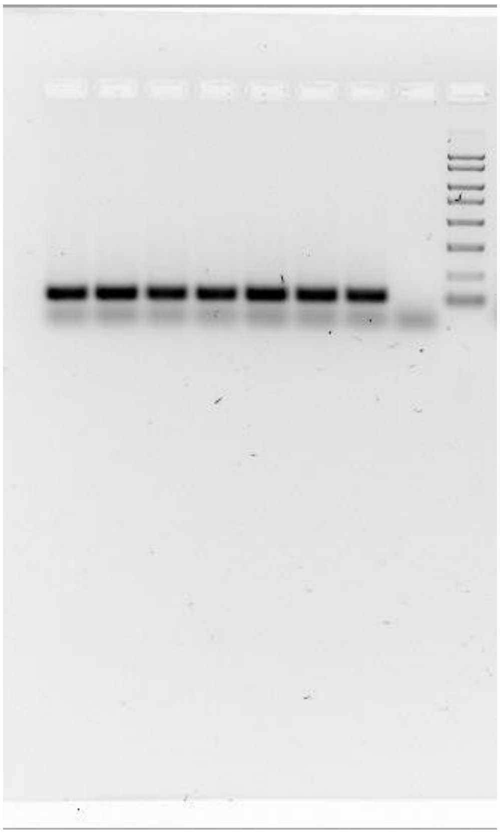

Supplement: Supplementary file 7 — Additional file 7. Fig. S7. Full gels and blots images [file 13058_2023_1686_MOESM7_ESM.pdf]
