# Supplementary material for: RAGE inhibition blunts insulin-induced oncogenic signals in breast cancer
Source: Breast Cancer Res. 2023 Jul 17;25:84. doi: 10.1186/s13058-023-01686-5 (PMC10351154; doi:10.1186/s13058-023-01686-5)

**Table S1.** Schematic representation of pathways affected by RAGE inhibition in Ins-stimulated MCF-7 cells, as evidenced by pathway enrichment analysis of proteomic outputs.


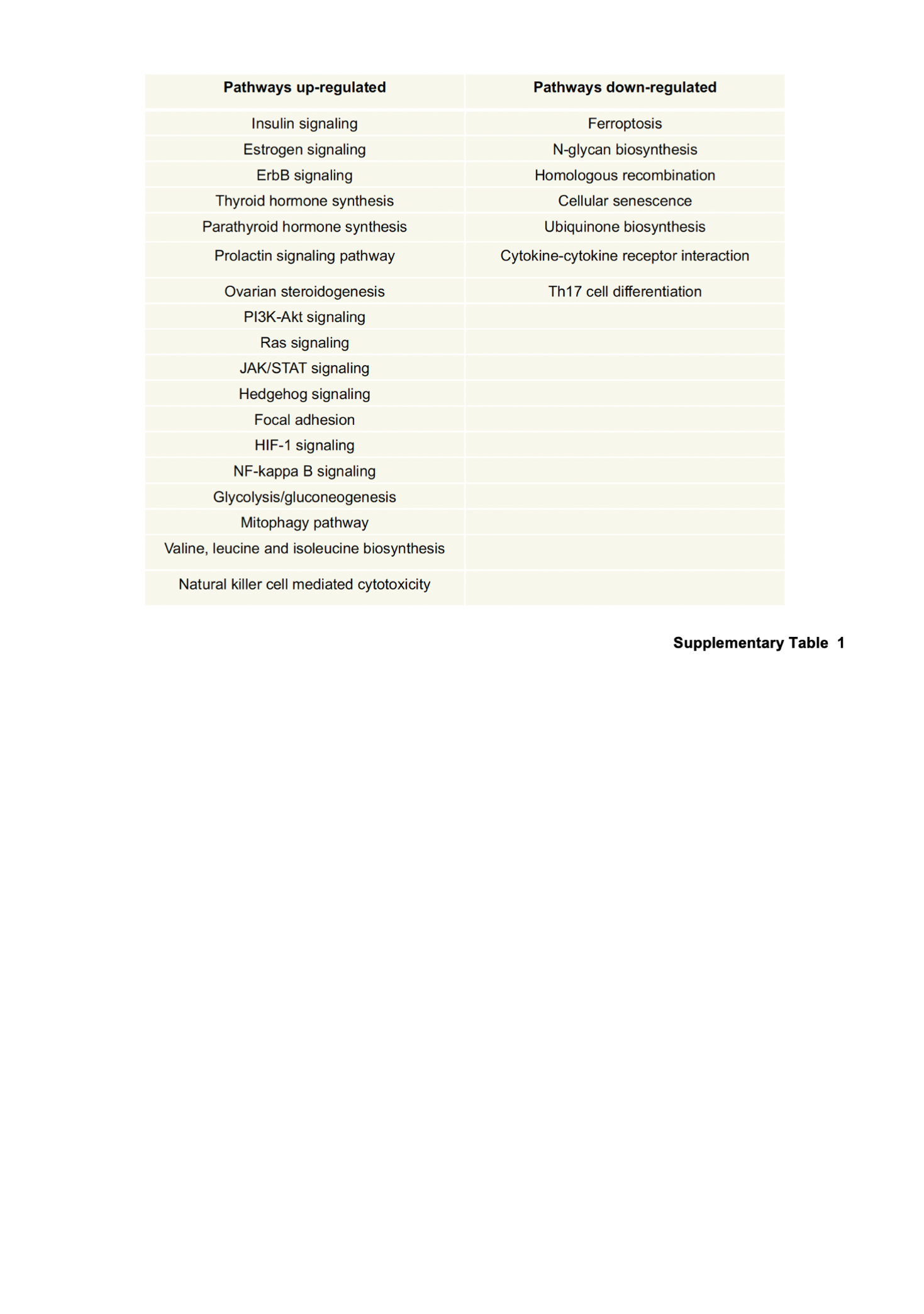

Supplement: Supplementary file 8 — Additional file 8. Table S1. Schematic representation of pathways affected by RAGE inhibition in Ins-stimulated MCF-7 cells, as evidenced by pathway enrichment analysis of proteomic outputs [file 13058_2023_1686_MOESM8_ESM.docx]
