# Supplementary material for: RAGE inhibition blunts insulin-induced oncogenic signals in breast cancer
Source: Breast Cancer Res. 2023 Jul 17;25:84. doi: 10.1186/s13058-023-01686-5 (PMC10351154; doi:10.1186/s13058-023-01686-5)

**Table S2.** Relevant clinical information and characteristics of patient-derived tumors used in the study.


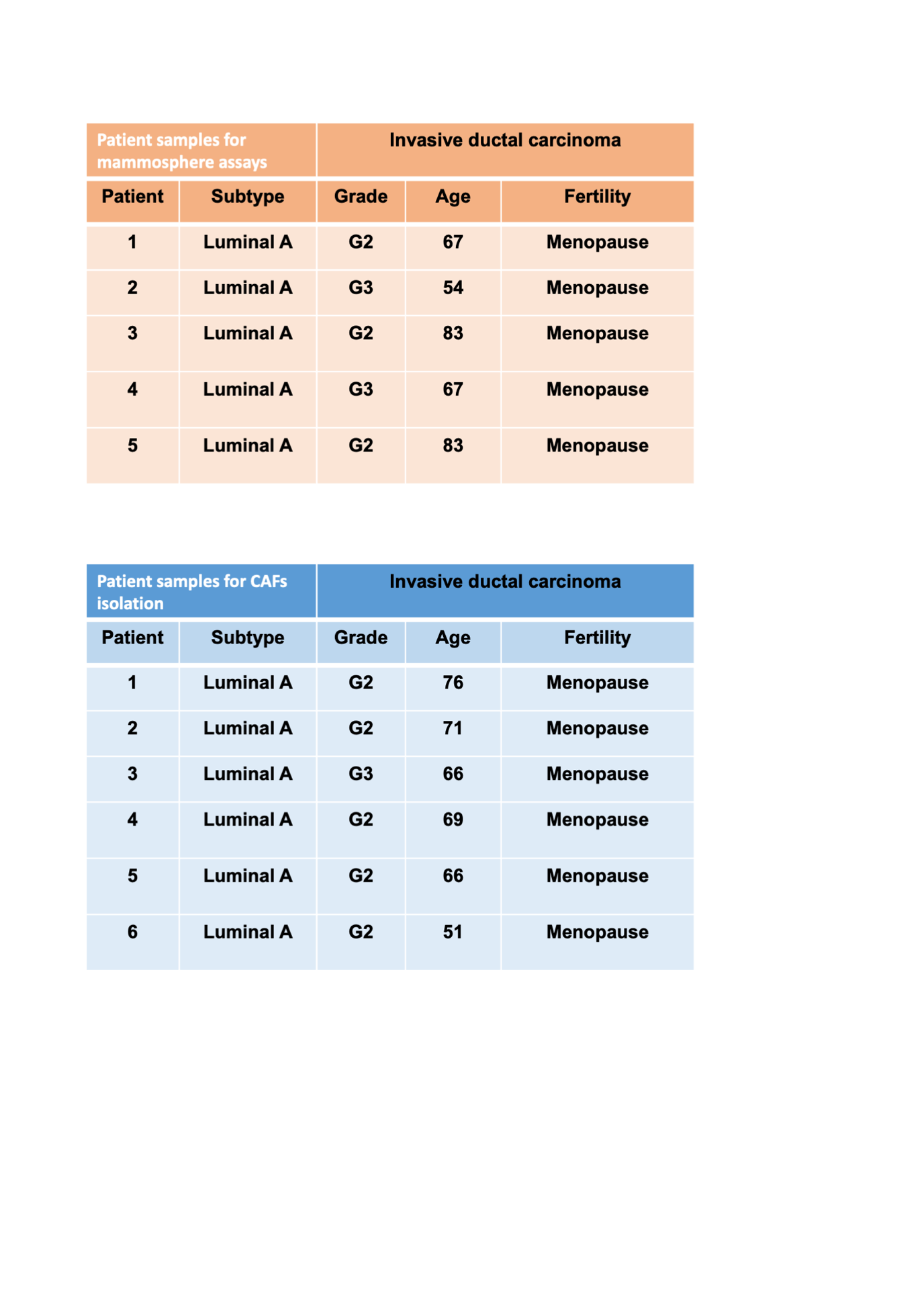

Supplement: Supplementary file 9 — Additional file 9. Table S2. Relevant clinical information and characteristics of patient-derived tumors used in the study [file 13058_2023_1686_MOESM9_ESM.docx]
