# Supplementary material for: RAGE inhibition blunts insulin-induced oncogenic signals in breast cancer
Source: Breast Cancer Res. 2023 Jul 17;25:84. doi: 10.1186/s13058-023-01686-5 (PMC10351154; doi:10.1186/s13058-023-01686-5)
Supplement: Supplementary file 10 — Additional file 10. Supplementary Materials and Methods. [file 13058_2023_1686_MOESM10_ESM.docx]

**Additional file 10 Supplementary Materials and Methods**

**Title page**

**RAGE inhibition blunts Insulin-induced oncogenic signals in breast cancer.**

Muoio MG^1,2^*, Pellegrino M^2^*, Rapicavoli V^1^, Talia M^2^, Scavo G^1^, Sergi V^1^, Vella V^1^, Pettinato S^3^, Galasso MG^4^, Lappano R^2^, Scordamaglia D^2^, Cirillo F^2^, Pulvirenti A^5^, Rigiracciolo DC^6^, Maggiolini M^2^, Belfiore A^1#^, De Francesco EM^1#^.

^1^Endocrinology, Department of Clinical and Experimental Medicine, University of Catania, Garibaldi-Nesima Hospital, 95122, Catania, Italy;

^2^Department of Pharmacy, Health and Nutritional Sciences, University of Calabria, 87036, Rende, Italy;

^3^Breast Unit Breast Surgery, Garibaldi-Nesima Hospital, 95122, Catania, Italy;

^4^Pathological Anatomy Unit, Garibaldi-Nesima Hospital, 95122, Catania, Italy;

^5^Bioinformatics Unit, Department of Clinical and Experimental Medicine, University of Catania, 95131 Catania, Italy

^6^Department of Experimental Oncology, IEO, European Institute of Oncology IRCCS, Via Adamello 16, 20139 Milano, Italy.

(*), (#) equal contribution

Correspondence to: Ernestina De Francesco; [ernestina.defrancesco@unict.it](mailto:ernestina.defrancesco@unict.it)

ORCID-EDF: 0000-0002-2810-6128;

Marcello Maggiolini; marcello.maggiolini@unical.it

**Acknowledgments:** The research leading to these results has received funding from AIRC under Start-Up 2018 – ID. 21651 – P.I. De Francesco Ernestina Marianna; AB was supported by Fondazione AIRC (IG 23369) by MUR under PNRR M4C2I1.3 Heal Italia project PE00000019 CUP B73C22001250006 (of University of Catania). AB and RL were supported by Ministero della Salute, Italy, grant RF-2019-12368937. AB, MM and RL were supported by Fondazione AIRC (IG 23369 to AB, IG 21322 to MM and IG27386 to RL); MM and RL acknowledge (i) the special award—namely, ‘Department of Excellence 2018–2022’ (Italian Law 232/2016)—to the Department of Pharmacy, Health, and Nutritional Sciences of the University of Calabria (Italy); (ii) PON Ricerca e Competitività 2007–2013 and the ‘Sistema Integrato di Laboratori per L’Ambiente—(SILA) PONa3_00341).

We wish to thank Dr. Duncan Smith (The University of Manchester) for his help with proteomics analyses and Dr. Maria Rizzotto (Garibaldi-Nesima Hospital) for assisting us in liaising with patients. We are very grateful to the patients who generously donated samples for our research. Illustrative figures were created using Biorender.

### Supplementary materials and methods

**CAFs isolation, cultivation and characterization.** BC surgical specimens were cut into smaller pieces (1 to 2 mm diameter), placed in digestion solution (400 IU collagenase, 100 IU hyaluronidase containing 10% FBS, antibiotics and antimycotics solution) and incubated overnight at 37°C. Following digestion, cells were separated by differential centrifugation at 90×g for 2 min; the supernatant obtained was centrifuged at 485×g for 8 min, while the pellet obtained was suspended in fibroblasts growth medium (Medium 199 and Ham’s F12 mixed 1:1 and supplemented with 10% FBS and 1% P/S) and cultured at 37°C, 5% CO_2_. CAFs passaged for up to five population doublings were used for subsequent experiments to minimize clonal selection and culture stress, which could occur during extended tissue culture. Primary cells cultures of breast fibroblasts were characterized by evaluating the gene expression of the epithelial marker E-cadherin and the mesenchymal marker Vimentin by RT-PCR studies. In order to assess fibroblasts activation, we evaluated the gene and protein expression of fibroblast activated protein α (FAPα) (by RT-PCR and immunofluorescence respectively). For classic RT-PCR analysis, the DreamTaq™ Hot Start PCR Master Mix kit (Thermo Fisher Scientific, Life Technologies, Italy) was used, in combination with the following primer pairs: hE-Cadherin Fwd: 5'- TGC CCA GAA AAT GAA AAA GG-3'; hE-Cadherin Rv 5’: 5'-GTG TAT GTG GCA ATG CGT TC-3'; hVimentin Fwd: 5'- GCG CCA GGC CCA AGC AGG AGT C-3'; hVimentin Rv: 5'-CCA GGG CCA TCT TAA CAT TGA GCA-3'; hFAPα Fwd 5’- TCC TGG CTT CAG CTT CCA AC-3’; hFAPα Rv 5’-TTT ACT CCC AAC AGG CGA CC-3’; hGAPDH Fwd: 5’- CAA GGC TGT GGG CAA GGT- 3’; hGAPDH Rv: 5’- GGA AGG CCA TGC CAG TGA-3’. Amplification products were resolved on 2% agarose gel and visualized using ChemiDoc Molecular Imager^®^ Gel Doc™ XR+ System with Image Lab™ Software (Bio-Rad, USA). For each sample GADPH expression served as a normalizing control. For the evaluation of FAP-α expression by immunofluorescence experiments.

**Lentiviral transduction.** Two days before transfection, 293Ta lentiviral packaging cells were transfected with lentiviral expression plasmid encoding for human RAGE (EX-A0070- Lv105-10 ORF expression), or empty vector (Ex-Neg, EX-NEG-Lv105 Empty control vector), using the Lenti-PacTM HIV Expression Packaging Kit, according to the manufacturer’s instructions. 48 h post transfection, lentivirus-containing culture medium was collected, centrifuged, filtered through a 0.45 μm PES filter, diluted in complete medium with 5μg/mL polybrene and then transferred to MCF-7 cells. Transduced MCF-7 cells were selected with 2.5 μg/mL puromycin (Thermo Fisher Scientific, Life Technologies, Italy).

**Gene expression studies.** Total RNA was extracted using TRIzol commercial kit (Thermo Fisher Scientific, Life Technologies, Italy), as recommended, quantified spectrophotometrically, and quality-checked by electrophoresis through agarose gels. Only samples that were not degraded and showed clear 18 and 28 S bands under UV light were used for RT-PCR. 2μg RNA was reverse-transcribed using the High capacity cDNA reverse transcription kit (Thermo Fisher Scientific, Life Technologies, Italy). Thereafter, the expression of selected genes was quantified by real-time PCR (ABI 7500 Real-Time PCR System, Applied Biosystems (Thermo Fisher Scientific, Life Technologies, Italy) with probe, primer sets and SYBR Green chemistry. mRNA quantification was performed using the comparative cycle threshold (CT) method (ΔΔCt). Primer sequences are as it follows: hCD1 Fwd 5′-TGC AAG GAA AAT TAG GGT ACT CA- 3’ and hCD1 Rv 5’- AGC CAA GAT GTG CAA ATT GT -3’; hALDH1A3 Fwd 5’-AGC AAG TAA GGG AGC GGA AA-3’ and hALDH1A3 Rv 5’- CCT TGC TAG CCC CTG AGA AG -3’; hRAGE Fwd 5’- GGA CCC TTA GCT GGC ACT TAG A- 3’; hRAGE Rv 5’- GAG TCC CGT CTC AGG GTG TCT – 3’; hIR Fwd 5’ – CGT GGA GGA TAA TTA CAT CGT GTT - 3’; hIR Rv 5’- TGG TCG GGC AAA CTT TCT G - 3’; hIGF-1R Fwd 5’- TGG TGG AGA ACG ACC ATA TCC - 3’ ; hIGF-1R Rv 5’- CGA TTA ACT GAG AAG AGG AGT TCG A -3’; h36B4 Fwd 5’- GCA GCA TCT ACA ACC CTG AAG TG -3’; h36B4 Rv 5’- TCC AGG AAG CGA GAA TGC A -3’. Assays were performed in duplicate, the results were normalized for 36B4 expression and then calculated as fold induction of RNA expression.

**Western Blot Analysis.** Cells exposed to treatments were lysed in RIPA buffer with protease (Merck, Millipore, Italy) and phosphatase inhibitors (Thermo Fisher Scientific, Life Technologies, Italy). Samples were then centrifuged at 13.000 rpm for 10 min and protein concentrations were determined using BCA protein assay according to the manufacturer’s instructions (Thermo Fisher Scientific, Life Technologies, Italy). Equal amounts of whole-protein extract were resolved through a reducing SDS 7.5, 4-15% (*w*/*v*) polyacrylamide gel (Biorad, Italy), electroblotted onto a nitrocellulose membrane (VWR, Italy) and probed with primary antibodies against: pIGF-1R (Y1135/Y1136)/pIR /Y1150/1151; catalog #3024) (19H7), IGF-1R (catalog #3027S), IR (L55B10; catalog #3020), pAKT (S473) (D9E; catalog #4060), AKT (C67E7; catalog #4691), RAGE (D1A12, catalog #6996), (all purchased from Cell Signaling Technology, distributed by Euroclone, Italy), CD1 (A12; catalog #sc-8396), CYR-61(A-10, catalog #sc-374129), ERα (D-12, catalog #sc-8005) (all purchased from Santa Cruz Biotechnology, DBA, Italy). Proteins were detected by horseradish peroxidase-linked secondary anti-rabbit and anti-mouse IgG antibodies (Cell Signaling Technology, distributed by Euroclone, Italy) and revealed using the West Pico Chemiluminescent Substrate (Thermo Fisher Scientific, Life Technologies, Italy). Chemiluminescent signal was revealed on Amersham high performance chemiluminescence films (Hyperfilms Amersham, VWR, Italy), or using the LI-COR Odyssey 2800 (Li-COR Inc., USA) and the software ImageStudioLite (version 5.2). β-actin (Sigma Aldrich, Italy) served as loading control.

**Co- Immunoprecipitation (Co-IP) assay.** After stimulation with Ins, MCF7-RAGE cells were washed with PBS and lysed using RIPA buffer (Sigma-Aldrich, Italy), containing phosphatase inhibitors (Thermo Fisher Scientific, Life Technologies, Italy) and protease inhibitors (Merck, Millipore, Italy), NaCl 150 mM, NP-40 20% 0,5%, EGTA 1mM (all purchased by Sigma-Aldrich, Italy). Samples were then centrifuged at 13.000 rpm for 10 min, and protein concentrations were determined using Pierce BCA protein assay (Thermo Fisher Scientific, Life Technologies, Italy). 1 mg proteins was then incubated for 2 h with immunoprecipitation buffer supplemented with inhibitors, 2 μg of anti-IR antibody (L55B10, catalog #3020) (Cell Signaling Technology, distributed by Euroclone, Italy) or non- specific IgG (I4506) (Sigma-Aldrich, Italy) and Protein G sepharose (Sigma-Aldrich, Italy). Samples were then centrifuged at 6.000 rpm for 1 min at 4°C and separated to pellet beads. Pellets were washed three times with 1 ml of RIPA buffer and then incubated overnight at 4°C. The following day, samples were centrifuged at 6.000 rpm for 1 min at 4°C and then resuspended in RIPA buffer with protease inhibitors, 2X SDS sample buffer and heated to 90 °C for 5 min. Samples were then processed as Western Blotting, according to standard procedures, as previously described.

***In situ* proximity ligation assay (PLA).** PLA was performed using the Duolink kit (Sigma-Aldrich, Italy) to detect IR and RAGE direct interaction using mouse anti-IR antibody (Cell Signaling Technology, distributed by Euroclone, Italy) and rabbit anti-RAGE antibody (Cell Signaling Technology, distributed by Euroclone, Italy), as recommended by the manufacturer. Briefly, fifty percent confluent MCF7-RAGE cells were plated onto LabTeK chamber slides (VWR, Italy), serum starved for 8 h and then stimulated with vehicle or Ins for 5 min. Slides were fixed with 4% paraformaldehyde (PFA), permeabilized with 0,2% Triton X-100 and after blocking, incubated with a rabbit primary antibody against RAGE (D1A12), (1:100) (Cell Signaling Technology, distributed by Euroclone, Italy) and mouse primary antibody against IR (L55B10), (1:100) (Cell Signaling Technology, distributed by Euroclone, Italy), overnight at 4°C. The following day, slides were washed twice with Wash Buffer A and incubated for 1 h with Anti-Rabbit PLUS probe and Anti-Mouse MINUS probe (PLA probes, 1:5), according to manufacturer’s instruction. Annealing of the PLUS and MINUS PLA probes occurs when IR and RAGE are in close proximity (< 30–40 nm), therefore repeat sequences in the annealed oligonucleotide complexes are amplified and then recognized by a fluorescently labeled oligonucleotide probe. PLA signals were detected using a fluorescence microscope TI-E (Nikon, Netherland).

**Unbiased label-free semi-quantitative proteomics and pathway analysis.** 4,5 x 10^6^ MCF-7 cells were s seeded in 150 mm petri dishes in regular growth medium. 24 h after seeding, medium was switched to 1% CT and subjected to treatments. Thereafter, proteins were collected using RIPA buffer (Sigma-Aldrich, Italy) devoid of protease and phosphatase inhibitors and supplemented with benzonase (Merck-Millipore 70664-10KUN) at 1 μl per 50 μl lysis buffer. Samples were stored on ice for 10 mins with pipetting every 1 min to aid full lysis. After centrifugation, supernatants were collected and samples were flash-frozen, after collecting small aliquots for protein quantification. Next, protein lysates were prepared for trypsin digestion by sequential reduction of disulfide bonds with TCEP and alkylation with MMTS. Then, peptides were extracted and prepared for LC-MS/MS analyses, which were performed on an LTQ Orbitrap XL mass spectrometer (Thermo Fisher Scientific, Life Technology, USA) coupled to an Ultimate 3000 RSLCnano system (Thermo Fisher Scientific, Life Technology, USA). Xcalibur raw data files acquired on the LTQ-Orbitrap XL were directly imported into Progenesis LCMS software (Waters Corp., UK) for peak detection and alignment. Five replicates were analyzed for each sample type. For statistical and differential expression protein (DEPs) analyses, data were imported into Rstudio (R V 1.2.5033). DEPs were identified using the LIMMA package (Bioconductor) [1]. We considered as differentially expressed all proteins with an absolute Log2FC > 0.6 and a p-value ≤ 0.05, as calculated by ANOVA. Pathway analysis was performed using the MITHrIL algorithm [2]. The underlying pathway topologies, composed of genes and their directional interactions, were obtained from Kyoto Encyclopedia of Genes and Genomes (KEGG) database [3]. Pathways were considered dysregulated if they had a corrected accumulator ≠ 0 and p-value < 0.05.

**Histologic analysis and immunohistochemistry.** Formalin-fixed, paraffin-embedded (FFPE) sections of tumor allografts were cut at 5 μm, mounted on slides precoated with poly-lysine, air dried, deparaffinized, rehydrated, (7–8 serial sections) and stained with hematoxylin and counterstained with eosin Y (Bio-Optica, Italy). Immunohistochemical experiments were performed after heat-mediated antigen retrieval. Hydrogen peroxide was used for 30 min, to inhibit endogenous peroxidase activity while normal goat serum (10%) was utilized, for 30 min, to block the non-specific binding sites. Morphologic analysis was carried out using hematoxylin and eosin standard staining. For the evaluation of CD1 expression, sections were incubated with a primary anti-human CD1 (Santa Cruz Biotechnology, DBA, Italy) antibody at 4°C overnight. Then, a universal biotinylated IgG was applied for 1 hour at room temperature, followed by ABC/HRP. Immunoreactivity was visualized by using DAB. For negative controls, nonimmune serum replaced at the same concentration of the primary antibody. Sections nuclei were counterstained with hematoxylin. For each sample six-seven serial sections were processed and visualized using an OPTIKA XDS-3 microscope (OPTIKA Microscopes, Italy) and the images were acquired with OpticalSview software using an OPTIKA 4883.13 CAM.

**References**

1. Ritchie M, Phipson B, Wu D et al (2015) Limma powers differential expression analyses for RNA-sequencing and microarray studies. Nucleic Acids Research 43(7):e47–e47. https://doi.org/10.1093/nar/gkv007

2. Alaimo S, Marceca GP, Ferro A et al (2017) Detecting Disease Specific Pathway Substructures through an Integrated Systems Biology Approach. Noncoding RNA 3(2):20. https://doi.org/10.3390/ncrna3020020

3. Kanehisa M, Goto S (2000) KEGG: Kyoto Encyclopedia of Genes and Genomes. Nucleic Acids Research 28(1):27–30. https://doi.org/10.1093/nar/28.1.27
